# Hyperbolic enhancement of photocurrent patterns in minimally twisted bilayer graphene

**DOI:** 10.1038/s41467-021-21792-2

**Published:** 2021-03-12

**Authors:** S. S. Sunku, D. Halbertal, T. Stauber, S. Chen, A. S. McLeod, A. Rikhter, M. E. Berkowitz, C. F. B. Lo, D. E. Gonzalez-Acevedo, J. C. Hone, C. R. Dean, M. M. Fogler, D. N. Basov

**Affiliations:** 1 Department of Physics, Columbia University, New York, NY 10027 USA; 2 Department of Applied Physics and Applied Mathematics, Columbia University, New York, NY 10027 USA; 3 Departamento de Teoría y Simulación de Materiales, Instituto de Ciencia de Materiales de Madrid, CSIC, Madrid, 28049 Spain; 4 Department of Physics, University of California, San Diego, La Jolla, CA 92093 USA; 5 Department of Mechanical Engineering, Columbia University, New York, NY 10027 USA

**Keywords:** Electronic properties and devices, Optical properties and devices

## Abstract

Quasi-periodic moiré patterns and their effect on electronic properties of twisted bilayer graphene have been intensely studied. At small twist angle *θ*, due to atomic reconstruction, the moiré superlattice morphs into a network of narrow domain walls separating micron-scale AB and BA stacking regions. We use scanning probe photocurrent imaging to resolve nanoscale variations of the Seebeck coefficient occurring at these domain walls. The observed features become enhanced in a range of mid-infrared frequencies where the hexagonal boron nitride substrate is optically hyperbolic. Our results illustrate the capabilities of the nano-photocurrent technique for probing nanoscale electronic inhomogeneities in two-dimensional materials.

## Abstract (web summary)

Here, the authors use scanning probe photocurrent imaging to resolve nanoscale variations of the Seebeck coefficient occurring at domain walls separating micron-scale AB and BA stacking regions in twisted bilayer graphene, and observe hyperbolic enhancement of the photocurrent pattern.

## Introduction

Twisted bilayer graphene (TBG), consisting of two graphene sheets rotated with respect to each other, has emerged as a tunable platform for studying exotic electronic phases. Transport experiments have revealed that when the graphene layers are twisted by a magic angle of *θ* ∼ 1.1°, TBG can become a superconductor^1^, a correlated insulator^2^, or a quantum anomalous Hall insulator^3–5^. A key feature of TBG is the moiré superlattice: a long-range variation in the atomic stacking arising from geometric interference of the lattice periodicities in the two graphene sheets. Scanning probe studies of TBG with *θ* ∼ 1.1° demonstrated spatial variations in the electronic properties occurring on the length scale of tens of nanometers^6–9^.

In minimally twisted bilayer graphene (MTBG), the moiré pattern periodicity is large, e.g., 140 nm for *θ* ≈ 0.01° and prone to atomic relaxation. In the relaxed state, the Bernal stacked domains (AB and BA) dominate while the less stable stacking configurations are reduced to a network of narrow domain walls (DWs). Transmission electron microscopy (TEM) measurements have shown that the DWs are 6–9 nm wide^10^. Previous transport^11^, nano-infrared^12,13^, and scanning tunneling microscopy (STM)^14^ studies have revealed the existence of topological states at the DWs when an electronic bandgap is opened by a sufficiently large interlayer bias between the graphene sheets. At smaller interlayer biases, the change in the atomic stacking across the DW still leads to a change in the electronic properties.

Scanning nano-photocurrent imaging has emerged as a novel optoelectronic probe capable of resolving changes in DC transport properties of graphene with nanometer-scale spatial resolution^15^. Previous nano-photocurrent experiments have resolved charge inhomogeneities and grain boundaries in monolayer graphene^15^ and mapped variations in twist angle of TBG at twist angles *θ* > 1°^16^. Here, we use scanning nano-photocurrent imaging to study domain walls in MTBG. We show that the photocurrent patterns arise from DC Seebeck coefficient variations occurring at the DWs on a nanometer length scale. We further propose and demonstrate a mechanism that utilizes the intrinsic hyperbolicity of the hBN substrate to enhance the DW features in photocurrent images.

## Results and discussion

**Fig. 1:**
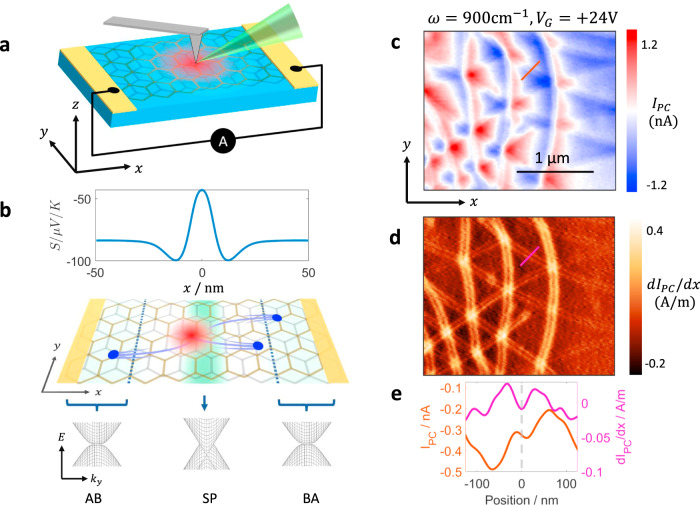
Photocurrent in minimally twisted bilayer graphene. **a** A schematic of scanning photocurrent setup. The red region represents the hot carriers generated under the tip. **b** Top: Seebeck coefficient *S* profile across a domain wall calculated from first principles (Supplementary Note 3). The DW is located at *x* = 0. Middle: perspective view of the experiment showing photocurrent generation at the domain wall. The green background represents the Seebeck coefficient profile and the blue dots represent carriers generated by thermoelectric effect. Bottom: schematic of the BLG band structure across the DW for three different stackings AB, BA, and saddle point (SP). **c** Photocurrent (*I*_PC_) image taken with *ω* = 900 cm^−1^ and $V_G = + 24\,{\mathrm{V}}$ at $T = 300\,{\mathrm{K}}$. **d** Spatial gradient of the photocurrent defined as d*I*_PC_/d*x* of the data in **c**. **e** Line profiles of *I*_PC_ and d*I*_PC_/d*x* across a DW (shown as red and magenta lines in **c** and **d**).

Figure 1(a) shows a schematic of our experiment. Infrared light is focused onto the apex of a sharp metallic tip, which enhances the electric field underneath the tip. The enhanced field locally generates a photocurrent, which we collect through electrical contacts at zero bias. In graphene, the photocurrent arises from electronic inhomogeneities through the photothermoelectric effect (PTE), schematically shown in Fig. 1(b)^17–19^. Photocurrent images are acquired by raster scanning the tip across the sample. Our technique overcomes the diffraction limit and provides a spatial resolution of about 20 nm while also allowing for simultaneous nano-infrared imaging^15^. Our device consists of two graphene layers with a minimal relative twist encapsulated between 37 nm bottom hexagonal boron nitride (hBN) layer and 6 nm top hBN layer. The entire stack rests on a 285 nm SiO_2_/Si substrate with the SiO_2_ layer serving as the gate dielectric. Piezoresponse force microscopy (PFM)^20^ before encapsulation of the device revealed domain walls with a periodicity of about 500 nm (Supplementary Note 1).

Figure 1(c) shows a representative photocurrent image of our device acquired at room temperature with laser frequency of *ω* = 900 cm^−1^. We use a color scheme that enables easy identification of the sign of the photocurrent: red and blue represent positive and negative currents respectively while white represents regions where the measured current is zero, thus highlighting the zero-crossing contours. Some of the zero-crossing contours form easily identifiable lines in the *y*-direction while others form a meandering pattern. On closer inspection, we find a series of fine structures in the photocurrent image that form a hexagonal lattice. These features are more clearly revealed in the map of the photocurrent gradient, d*I*_PC_/d*x*, shown in Fig. 1(d). The periodicity of these features is consistent with the domain walls observed in PFM images before encapsulation (Supplementary Note 1). In the d*I*_PC_/d*x* image, the vertical domain walls appear to be more intense because of the contact configuration used in our experiments, as explained in Supplementary Note 3.1. The lattice structure and the matching periodicity lead us to conclude that the fine features correspond to the domain walls of a relaxed moiré superlattice in TBG.

**Fig. 2:**
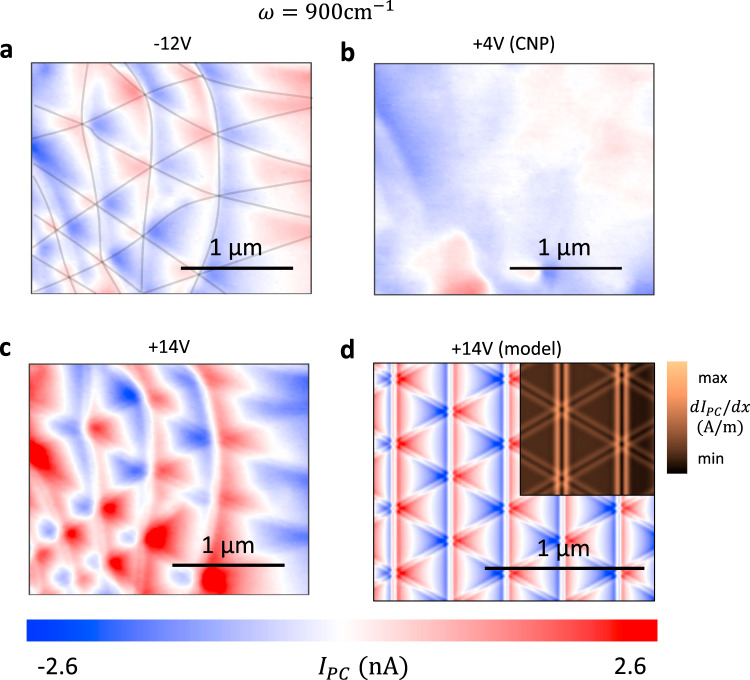
Thermoelectric origin of the photocurrent in TBG. **a**–**c** Gate voltage dependence of the photocurrent (*I*_PC_) at *ω* = 900 cm^−1^ and $T = 300{\mathrm{K}}$. Gate voltage is indicated above each panel. **d** Calculated photocurrent pattern using the Shockley–Ramo formalism^24^ with material parameters corresponding to $V_G = + 14{\mathrm{V}}$ (more details in Supplementary Note 3). The top-right inset shows the calculated image of d*I*_PC_/d*x* (compare with Fig. 1(d)).

Next, we study the gate dependence of the photocurrent maps as plotted in Fig. 2(a–c). Transport experiments on our device showed that the charge neutrality point (CNP), where the carrier density is minimal and the majority carriers change from holes to electrons, occurs at *V*_*G*_ = +4V (Supplementary Note 1). Photocurrent imaging at the CNP (Fig. 2(b)) does not show any of the features observed in Fig. 1(c). A comparison of the images at $V_G = - 12\,{\mathrm{V}}$ (Fig. 2(a)) and $V_G = + 14\,{\mathrm{V}}$ (Fig. 2(c)) reveals that the photocurrent has identical meandering pattern and fine DW features for positive and negative gate voltages except for a sign change. These results show that the meandering patterns and the DW features are antisymmetric with respect to the carrier type. As the gate voltage increases further in both the positive and negative direction, we find that the patterns weaken and eventually become unresolvable (Supplementary Note 2). We note that the carrier densities in Fig. 2 are too low to produce significant plasmonic effects in bilayer graphene (Supplementary Note 1.2).

Previous theoretical^19^ and experimental^18,21,22^ investigations have discovered that the dominant mechanism for photocurrent generation in graphene is the photothermoelectric effect (PTE). In this mechanism, the absorption of incident light generates hot carriers in graphene. When the hot carriers encounter variations in the Seebeck coefficient, a thermoelectric voltage is generated, which drives a current through the sample. The spatial profile of the measured current is, therefore, directly related to the Seebeck coefficient profile in the sample. PTE shows several characteristic features in experiments. First, since Seebeck coefficient is antisymmetric with respect to the sign of the carriers, the resulting photocurrent patterns also change sign when the carrier type changes from holes to electrons^18,21^. Second, the Seebeck coefficient of bilayer graphene rapidly diminishes as the carrier density increases^21,23^. Therefore, any variations in the Seebeck coefficient and the resulting photocurrent must also become small. Both features are present in our data, strongly suggesting that the photocurrent patterns we observe arise from PTE.

To confirm our hypothesis that the photocurrent arises from PTE and to gain a deeper understanding of our results, we calculated the expected photocurrent patterns from PTE. The input to these calculations are the Seebeck coefficient profile and the hot carrier temperature profile. We computed the former for an isolated domain wall using a generalized Boltzmann approach (Supplementary Note 3) and the resulting profile is shown in Fig. 1(b). To compare with our experiment, we superposed the one-dimensional Seebeck profiles in a hexagonal pattern to generate a two-dimensional lattice of domain walls (Supplementary Note 3.4). Next, we computed the spatial profile of the hot carriers. We first computed the electric field at the graphene surface using two different models for the tip^1^ a “lightning-rod model” in which the tip is represented by a conducting hyperboloid and^2^ a simplified common approximation of the tip by a vertically oriented point dipole (Supplementary Note 3.3 and 3.4). Since the conductivity of the graphene sheet is dominated by the in-plane components, we assumed that the radially symmetric in-plane field, *E*_*r*_, governs the generation of hot carriers. We then solved the heat equation to determine the spatial profile of the hot carrier temperature (Supplementary Note 3.1).

The Seebeck coefficient profile and the electron temperature profile are sufficient to calculate the local thermoelectric voltage for a given tip position. For gapless materials such as graphene, the photocurrent collected by distant electrodes also depends on the contact geometry. We used the Shockley–Ramo formalism of ref. ^24^ to include the effects of the contacts and our calculation procedures are described in more detail in Supplementary Note 3.

The photocurrent pattern resulting from the hyperboloid tip calculation is shown in Fig. 2(d). Our results reproduce the key features of our data including the meandering patterns and the fine features at the domain walls. We can now correlate the features in the photocurrent images with those in the Seebeck coefficient. The fine features and the zero-crossing contours that form straight lines along the *y*-axis arise from the domain walls themselves. On the other hand, the meandering zero-crossing contours go across domain walls, and arise from the interference of photocurrents generated by neighboring domain walls. The good agreement between calculations and data confirms that our photocurrent experiments directly probe the nanometer-scale Seebeck coefficient variations present at the domain walls.

While the first-principles Seebeck coefficient profile produced a photocurrent pattern similar to the experiment, we note that our experiment is not sensitive to the fine details of the Seebeck coefficient at the domain wall. In fact, any change in Seebeck on a length scale significantly shorter than the spatial extent of the hot carriers (typically called the cooling length^15^) will produce a pattern similar to the experiment, as we demonstrate in Supplementary Note 3.3.

So far, the hBN layers that surround the graphene sheet have not played an active role. We now show that the optical properties of hBN can be exploited to enhance the photocurrent features from the DWs. Over two frequency bands in the mid-infrared, referred to as the lower and upper Reststrahlen bands, the permittivity of hBN along its in-plane and out-of-plane principal axes have opposite signs^25^. Such behavior, known as hyperbolicity, leads to highly confined phonon polaritons^25–29^ and hyperlensing effects^30,31^. Here, we specifically focus on the upper Reststrahlen band (1376 to 1614 cm^−1^), where hBN transverse dielectric constant in the *xy*-plane becomes negative (${\it{\epsilon }}_t < 0$). The out-of-plane dielectric constant remains positive (${\it{\epsilon }}_z > 0$) and is weakly frequency dependent.

**Fig. 3:**
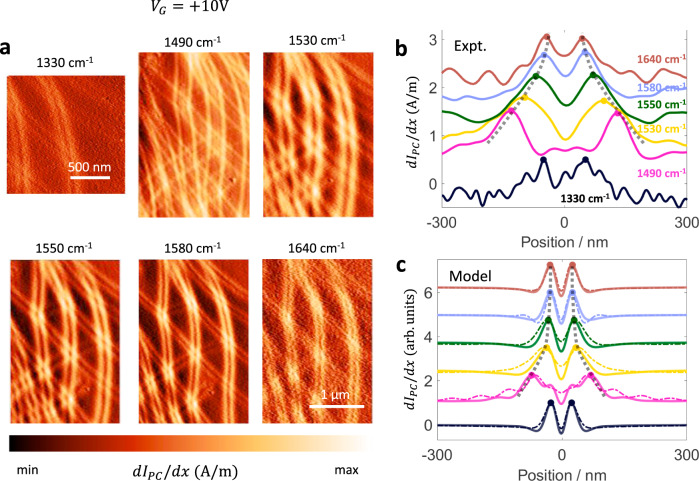
Domain wall photocurrent patterns in the hBN Reststrahlen band. **a** Gradient of photocurrent (d*I*_PC_/d*x*) for several frequencies around the hBN Reststrahlen band. **b** Experimental line profiles of d*I*_PC_/d*x* for several frequencies. The black dashed lines are guides to the eye. **c** Photocurrent profiles calculated using the frequency-dependent electric field profiles. The thick solid lines correspond to the hyperboloid tip and the thin dashed lines correspond to the point dipole model. The theoretical curves are normalized to their respective maxima. Curves in panel **b** and **c** are offset vertically for clarity.

We performed photocurrent experiments at several frequencies around the upper Reststrahlen band and the data is shown in Fig. 3(a). We observe a clear change in the width of the domain wall feature with frequency. Specifically, we find that at the lower end of the Reststrahlen band (e.g., *ω* = 1490 cm^−1^ and *ω* = 1530 cm^−1^ in Fig. 3(a)) the domain wall feature is wider compared with pattern below the reststrahlen band (compare, for example, with *ω* = 900 cm^−1^ of Fig. 1(d)). As the frequency increases, the width of the broad features decreases. Finally, at frequencies above the Reststrahlen band (*ω* = 1640 cm^−1^ in Fig. 3(a)), the width of the feature returns to its value below the Reststrahlen band. Furthermore, at *ω* = 1490 cm^−1^, we observe two faint peaks in between the two stronger peaks. These effects are further confirmed by the frequency-dependent line profiles shown in Fig. 3(b). From the line profiles, we see that the fainter peaks at *ω* = 1490 cm^−1^ are approximately coincident with the original peaks at *ω* = 1330 cm^−1^ and 1640 cm^−1^.

Since our experiments at *ω* = 900 cm^−1^ and the related modeling have shown that the photocurrent pattern is of PTE origin, any change in the pattern must be due to either a change in the Seebeck coefficient profile or the hot carrier profile. The Seebeck coefficient is not expected to change with the frequency of light incident on the material in the linear regime and the laser power used in our experiment (~20 mW, see Supplementary Note 1) is too weak to produce a significant non-linear effect. Therefore, we are led to conclude that change in the hot carrier distribution must be responsible for the observed change in width.

The spatial profile of Joule heating power is determined by the electric field profile under the tip and the real part of the optical conductivity of bilayer graphene, ${\mathrm{Re}}(\sigma )$. The frequency dependence data of Fig. 3 was collected at $V_G = + 10{\mathrm{V}}$, where the estimated Fermi energy in the Bernal stacked regions is low ($E_F \approx 10{\mathrm{meV}}$, refer to Supplementary Note 1.2) and the optical conductivity is dominated by the frequency-independent interband conductivity^32,33^. Therefore, we conclude that the electric field profile under the tip must change with frequency within the Reststrahlen band in order to reproduce the experimental observations shown in Fig. 3. To model the observed change in width, we used the “lightning-rod” model and a point dipole model to compute the radial electric field at several frequencies around the Reststrahlen band (Supplementary Note 3.3 and 3.4). The photocurrent profiles from our modeling are shown in Fig. 3(c) and show good agreement with the experiment.

**Fig. 4:**
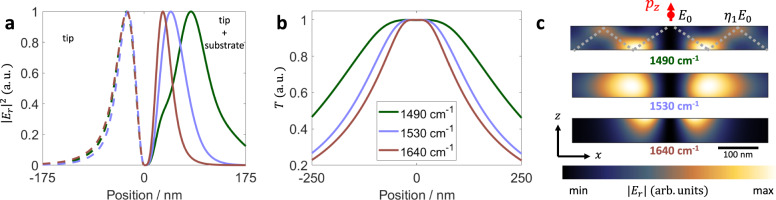
Local electric field and temperature inside and outside the Reststrahlen band. **a** In-plane electric field $\left| {E_r} \right|^2$ at the graphene layer calculated for a hyperboloid tip. The left half (dashed lines) shows the field of the tip alone and the right half (solid lines) shows the total field from the tip and the substrate. **b** Hot carrier temperature *T* calculated using the total field from **a**. **c** Cross section of a hBN slab showing the electric field resulting from excitation by a point dipole located above the hBN surface. 1490 and 1530 cm^−1^ are inside and 1640 cm^−1^ is outside the Reststrahlen band, respectively. The dashed line in the 1490 cm^−1^ image shows the polariton propagation. *E*_0_ and $\eta _1E_0$ represent the magnitude of the in-plane field at the zeroth order and the first order maxima, respectively.

The electric field at the graphene layer can be thought of as the sum of two separate parts. The first part is the incident field from the tip and the second part is the field reflected by the hBN substrate in response to the tip excitation. The left panels in Fig. 4(a) show the tip field and the right panels show the total field. We see that the tip field is weakly dependent on the frequency but the field reflected by the substrate is strongly modified inside the Reststrahlen band. The wider electric field leads to a wider hot carrier temperature profile (Fig. 4(b)) and a broader photocurrent pattern (Fig. 3(c)).

The origin of this widening is closely related to a previously observed effect in hBN slabs, known as hyperlensing^30,31,34^. In hyperlensing, a sub-wavelength antenna launches phonon polariton rays that propagate inside the hBN slab. Here, our tip acts as the antenna. The total in-plane field at the hBN surface can be thought of as a series of concentric rings centered below the tip with a radius of $r_k^{{\mathrm{max}}}$ for the *k*-th ring. The electric field at the *k*-th ring is given by $E_k = \eta _kE_0$, where $\eta _k$ is related to the permittivity of the hBN slab and the substrate (Supplementary Note 3.4). The *k* = 0 ring corresponds to the field from the tip itself with magnitude *E*_0_ while $k = 1,\,2, \ldots$ correspond to phonon polaritons propagating in the hBN slab (see Fig. 4(c) and Supplementary Note 3.4). Therefore, inside the Reststrahlen band, the zeroth order maximum is frequency independent but the higher order maxima are strongly frequency dependent.

Typically, the magnitude of the field at the *k* = 1 ring is expected to be smaller than the field created directly by the tip ($\left| {\eta _1} \right| < 1$). However, for several frequencies inside the Reststrahlen band, $\left| {\eta _1} \right| > 1$, so *E*_1_ dominates and leads to a broad frequency-dependent electric field profile and photocurrent pattern. The faint central features in d*I*_PC_/d*x* at *ω* = 1490 cm^−1^ can now be understood as arising from *E*_0_ while the stronger broader features arise from *E*_1_. In principle, the polariton reflections corresponding to *E*_2_ and higher order terms should be reflected in the photocurrent profile. Our simulations suggest that a sharper tip and more widely separated domain walls (i.e., smaller twist angle) could reveal such features in future photocurrent experiments (Supplementary Note 3.4).

In conclusion, we have demonstrated that nano-photocurrent experiments are sensitive to nanoscale changes in the Seebeck coefficient at the domain walls in MTBG. Our modeling of the photocurrent patterns is consistent with experiment. We further demonstrate a hyperbolic optoelectronic effect where the domain wall photocurrent patterns are enhanced by the hyperbolicity of the hBN substrate.

Note: While preparing our manuscript for submission, we became aware of a similar work by Hesp et al.^35^.

## Methods

### Device fabrication

The minimally twisted bilayer graphene device was fabricated using the dry transfer method. Piezoresponse force microscopy (PFM)^20^ was performed before encapsulation to ensure that a moiré pattern with a large periodicity was present (Supplementary Fig. 1(A)). The contact geometry was specifically designed for easy interpretation of photocurrent experiments (Supplementary Fig. 1(B), refer to Supplementary Note 3.1 on photocurrent modeling). We used the M1–M3 contacts for all photocurrent experiments.

### Bilayer graphene parameter estimate

The properties of bilayer graphene depend not only on the carrier density but also on the interlayer bias. In our experiment, we have a single Si back gate, which allows us to control the carrier density accurately. Here, we describe our estimate of the interlayer bias values for different gate voltages.

First, we assume that the interlayer bias is zero at charge neutrality point $V_G = + 4{\mathrm{V}}$. This assumption is reasonable for the ultra-high quality, doubly encapsulated devices studied in this work^36^. For a given gate voltage, we can directly calculate the displacement field below the graphene layers:

1 $$D_{{\mathrm{lower}}} = \frac{{{\it{\epsilon }}_{{\mathrm{lower}}}\,V_G}}{{d_{{\mathrm{lower}}}}}$$

where *∈*_lower_ and *d*_lower_ are the dielectric constant and thickness of the SiO_2_ dielectric layer. As we have no top gate, the displacement field above the graphene layers $D_{{\mathrm{upper}}} = 0$ and effective displacement field across the graphene is given by:

2 $$\bar D = \frac{{D_{{\mathrm{upper}}} + D_{{\mathrm{lower}}}}}{2} = \frac{{D_{{\mathrm{lower}}}}}{2}$$

We use ref. ^37^ to estimate the interlayer bias *V*_*i*_ from $\bar D$. To estimate *E*_F_, we keep *V*_*i*_ fixed and vary the Fermi energy *E*_F_ until the carrier density we calculate with a tight-binding model matches the value expected from capacitance calculations. Supplementary Fig. 2 shows a plot of the estimated *E*_F_ and *V*_*i*_ for several gate voltages. We find that the estimated Fermi energy is linear with gate voltage. At small displacement fields, the band structure of bilayer graphene can be well approximated to be parabolic^38^. In two dimensions, a parabolic dispersion leads to a constant density of states and a linear dependence of the Fermi energy on carrier density, which is consistent with our estimate. We note that the carrier densities considered in this manuscript (Figs. 2 and 3) are too low to produce significant plasmonic effects. In bilayer graphene, plasmons are typically observed in nano-infrared imaging for $V_G - V_{{\mathrm{CNP}}} > \sim 30{\mathrm{V}}$ ^39^.

### Nano-photocurrent experiments

Room temperature nano-photocurrent measurements were performed in a commercial s-SNOM from Neaspec GmbH. Low-temperature nano-photocurrent measurements were performed in a home-built SNOM within an ultra-high vacuum chamber^40^ at *T* = 200 K. For the *ω* = 900 cm^−1^ experiments, we used a CO_2_ laser and for the Reststrahlen band experiments, we used a tunable quantum cascade laser from Daylight Solutions. The incident laser power was around 20 mW in all cases. The current was measured using a Femto DHPCA-100 current amplifier. To isolate the photocurrent contributions from the near-fields localized under the tip, the measured current was demodulated at a harmonic *n* of the tapping frequency. In this work, we used *n* = 3 for room temperature experiments and *n* = 2 for low-temperature experiments.

## Supplementary information

Supplementary Information

## Data Availability

The datasets generated and analyzed during the current study are available from the corresponding author on reasonable request.

## Code availability

The computer code used for the analysis and simulations in the current study are available from the corresponding author on reasonable request.

## References

[1] Cao, Y. et al. Unconventional superconductivity in magic-angle graphene superlattices. *Nature* **556**, 1–17 (2018). 10.1038/nature26160 29512651

[2] Cao Y (2018). Correlated insulator behaviour at half-filling in magic-angle graphene superlattices. Nature.

[3] Sharpe AL (2019). Emergent ferromagnetism near three-quarters filling in twisted bilayer graphene. Science.

[4] Serlin M (2020). Intrinsic quantized anomalous Hall effect in a moiré heterostructure. Science.

[5] Nuckolls, K. P. et al. Strongly correlated Chern insulators in magic-angle twisted bilayer graphene. *Nature*10.1038/s41586-020-3028-8 (2020). 10.1038/s41586-020-3028-8 33318688

[6] Kerelsky A (2019). Maximized electron interactions at the magic angle in twisted bilayer graphene. Nature.

[7] Xie Y (2019). Spectroscopic signatures of many-body correlations in magic-angle twisted bilayer graphene. Nature.

[8] Jiang Y (2019). Charge order and broken rotational symmetry in magic-angle twisted bilayer graphene. Nature.

[9] Choi Y (2019). Electronic correlations in twisted bilayer graphene near the magic angle. Nat. Phys..

[10] Alden JS (2013). Strain solitons and topological defects in bilayer graphene. Proc. Natl Acad. Sci..

[11] Ju L (2015). Topological valley transport at bilayer graphene domain walls. Nature.

[12] Jiang BY (2017). Plasmon reflections by topological electronic boundaries in bilayer graphene. Nano Lett..

[13] Sunku SS (2018). Photonic crystals for nano-light in moiré graphene superlattices. Science.

[14] Yin LJ, Jiang H, Bin Qiao J, He L (2016). Direct imaging of topological edge states at a bilayer graphene domain wall. Nat. Commun..

[15] Woessner A (2016). Near-field photocurrent nanoscopy on bare and encapsulated graphene. Nat. Commun..

[16] Sunku SS (2020). Nano-photocurrent mapping of local electronic structure in twisted bilayer graphene. Nano Lett..

[17] Xu X, Gabor NM, Alden JS, van der Zande AM, McEuen PL (2010). Photo-thermoelectric effect at a graphene interface junction. Nano Lett..

[18] Gabor NM (2011). Hot carrier-assisted intrinsic photoresponse in graphene. Science.

[19] Song JCW, Rudner MS, Marcus CM, Levitov LS (2011). Hot carrier transport and photocurrent response in graphene. Nano Lett..

[20] McGilly LJ (2020). Visualization of moiré superlattices. Nat. Nanotechnol..

[21] Xu X, Gabor NM, Alden JS, Van Der Zande AM, McEuen PL (2010). Photo-thermoelectric effect at a graphene interface junction. Nano Lett..

[22] Cao H (2016). Photo-Nernst current in graphene. Nat. Phys..

[23] Zuev YM, Chang W, Kim P (2009). Thermoelectric and magnetothermoelectric transport measurements of graphene. Phys. Rev. Lett..

[24] Song JCW, Levitov LS (2014). Shockley-Ramo theorem and long-range photocurrent response in gapless materials. Phys. Rev. B..

[25] Caldwell JD (2014). Sub-diffractional volume-confined polaritons in the natural hyperbolic material hexagonal boron nitride. Nat. Commun..

[26] Dai S (2014). Tunable phonon polaritons in atomically thin van der Waals crystals of boron nitride. Science.

[27] Yoxall E (2015). Direct observation of ultraslow hyperbolic polariton propagation with negative phase velocity. Nat. Photon..

[28] Basov, D. N., Fogler, M. M. & García De Abajo, F. J. Polaritons in van der Waals materials. *Science* **354**, 10.1126/science.aag1992 (2016). 10.1126/science.aag1992 27738142

[29] Basov DN, Asenjo-Garcia A, Schuck PJ, Zhu X, Rubio A (2020). Polariton panorama. Nanophotonics.

[30] Dai S (2015). Subdiffractional focusing and guiding of polaritonic rays in a natural hyperbolic material. Nat. Commun..

[31] Li P (2015). Hyperbolic phonon-polaritons in boron nitride for near-field optical imaging and focusing. Nat. Commun..

[32] Nair RR (2008). Fine structure constant defines visual transparency of graphene. Science.

[33] Li ZQ (2008). Dirac charge dynamics in graphene by infrared spectroscopy. Nat. Phys..

[34] Woessner, A. et al. Electrical detection of hyperbolic phonon-polaritons in heterostructures of graphene and boron nitride. *NPJ 2D Mater. Appl*. **1**, 1–5 (2017).

[35] Hesp, N. C. H. et al. Nano-imaging photoresponse in a moiré unit cell of minimally twisted bilayer graphene. *Nat. Commun.*10.1038/s41467-021-21862-5 (2021). 10.1038/s41467-021-21862-5 PMC7954806 33712606

[36] Tan, C. et al. Realization of a universal hydrodynamic semiconductor in ultra-clean dual-gated bilayer graphene. Preprint at https://arxiv.org/abs/1908 (2019).

[37] Zhang Y (2009). Direct observation of a widely tunable bandgap in bilayer graphene. Nature.

[38] McCann, E. & Koshino, M. The electronic properties of bilayer graphene. *Reports Prog. Phys*. **76**10.1088/0034-4885/76/5/056503 (2013). 10.1088/0034-4885/76/5/056503 23604050

[39] Fei Z (2015). Tunneling plasmonics in bilayer graphene. Nano Lett..

[40] Post KW (2018). Coexisting first- and second-order electronic phase transitions in a correlated oxide. Nat. Phys..

