# Supplementary material for: Hyperbolic enhancement of photocurrent patterns in minimally twisted bilayer graphene
Source: Nat Commun. 2021 Mar 12;12:1641. doi: 10.1038/s41467-021-21792-2 (PMC7955135; doi:10.1038/s41467-021-21792-2)
Supplement: Supplementary file 1 — Supplementary Information [file 41467_2021_21792_MOESM1_ESM.pdf]

Supplementary Information for "Hyperbolic enhancement of photocurrent patterns in minimally twisted bilayer graphene"

S. S. Sunku[1,2,*], D. Halbertal[1,*,†], T. Stauber[3], S. Chen[1,^], A. S. McLeod[1], A. Rikhter[4], M. E. Berkowitz[1], C. F. B. Lo[1], D. E. Gonzalez-Acevedo[1,^], J. C. Hone[5], C. R. Dean[1], M. M. Fogler[4], D. N. Basov[1]

[1] Department of Physics, Columbia University, New York, NY

[2] Department of Applied Physics and Applied Mathematics, Columbia University, New York, NY

[3] ICMM at CSIC, Madrid, Spain

[4] Department of Physics, University of California, San Diego, La Jolla, CA

[5] Department of Mechanical Engineering, Columbia University, New York, NY

* These authors contributed equally

^ Present address: Department of Physics, Harvard University, Cambridge, MA

† dh2917@columbia.edu (D.H.)

Supplementary Note 1: Device characterization

Supplementary Figure 1 shows the piezoresponse force microscopy (PFM) (*1*) image of the graphene layers before encapsulation, the contact configuration used for photocurrent experiments and the determination of charge neutrality. Supplementary Figure 2 shows the dependence of the bilayer graphene parameters E_F and V_i on the applied gate voltage.

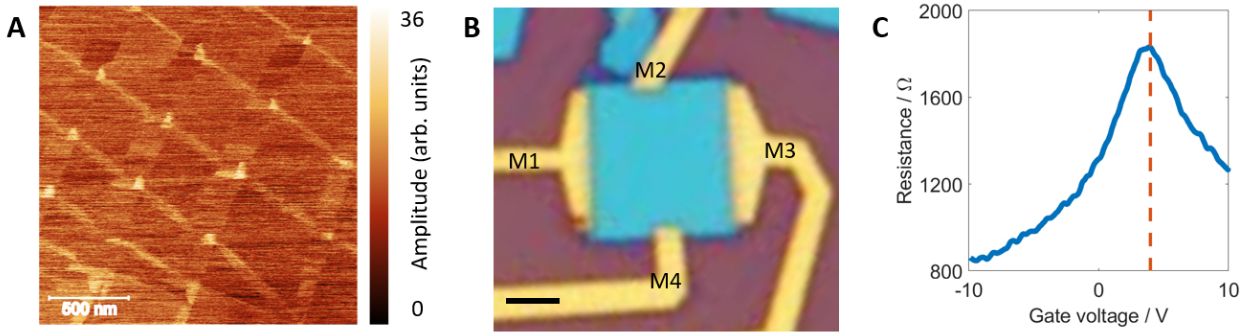

Supplementary Figure 1 | Device fabrication. (A) Piezoresponse force microscopy image of the graphene layers before encapsulation showing domain walls. (B) Optical microscope image showing the final contact configuration. Scale bar $3\mu\mathrm{m}$. (C) Two probe resistance measured using M1 and M3 contacts as a function of V_G applied to the Si back gate. The dashed line corresponds to $V_G = +4\mathrm{V}$ which is taken to be the charge neutrality point (Figure 2 (A) of main text).

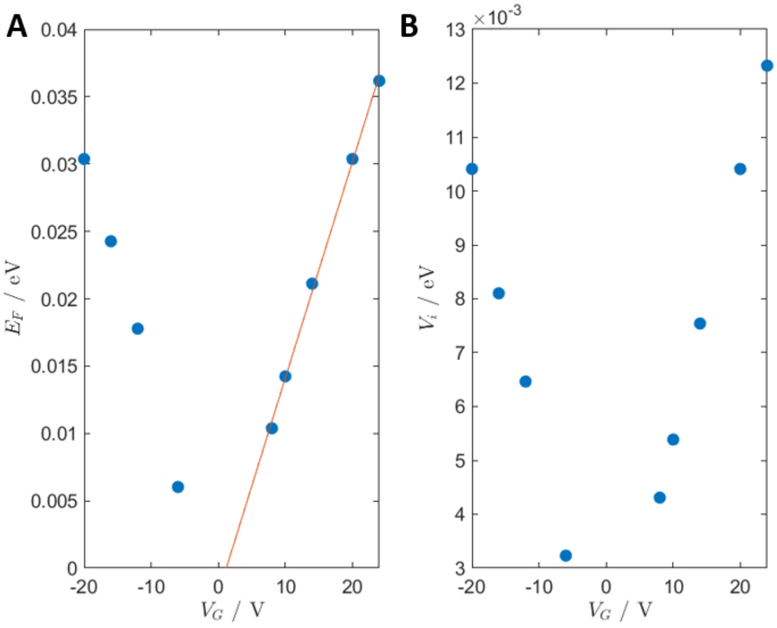

Supplementary Figure 2 | Estimated Fermi energy and interlayer bias for bilayer graphene with a single gate. The dots correspond to specific gate voltages and the red line is a linear fit.

Supplementary Note 2: More photocurrent data

Here, we describe our analysis methods for the photocurrent data and include all of the collected images. The photocurrent signal was demodulated at a harmonic of the tip tapping frequency with a lock-in amplifier. The phase offset of the demodulation signal is arbitrary since the phase only determines the direction of the current and otherwise does not contain any meaningful information. Therefore, for each photocurrent image, we adjusted the phase offset so as to maximize the signal in the in-phase component and minimize it in the out-of-phase component. Stated more rigorously, $S_{in}(x,y), S_{out}(x,y)$ are the raw data images for in-phase and out-of-phase lock-in output channels. For an offset phase ϕ_0, the corrected signal $S'_{in}(x,y), S'_{out}(x,y)$ is the result of rotation by ϕ_0:

$$\begin{pmatrix} S'_{in} \\ S'_{out} \end{pmatrix} = \begin{pmatrix} \cos\phi_0 & \sin\phi_0 \\ -\sin\phi_0 & \cos\phi_0 \end{pmatrix} \begin{pmatrix} S_{in} \\ S_{out} \end{pmatrix} \quad \text{(Eq 1)}$$

The offset angle ϕ_0 is chosen as to minimize the variance of S'_{out} across the image.

Supplementary Note 2.1: $\omega = 900\text{cm}^{-1}$

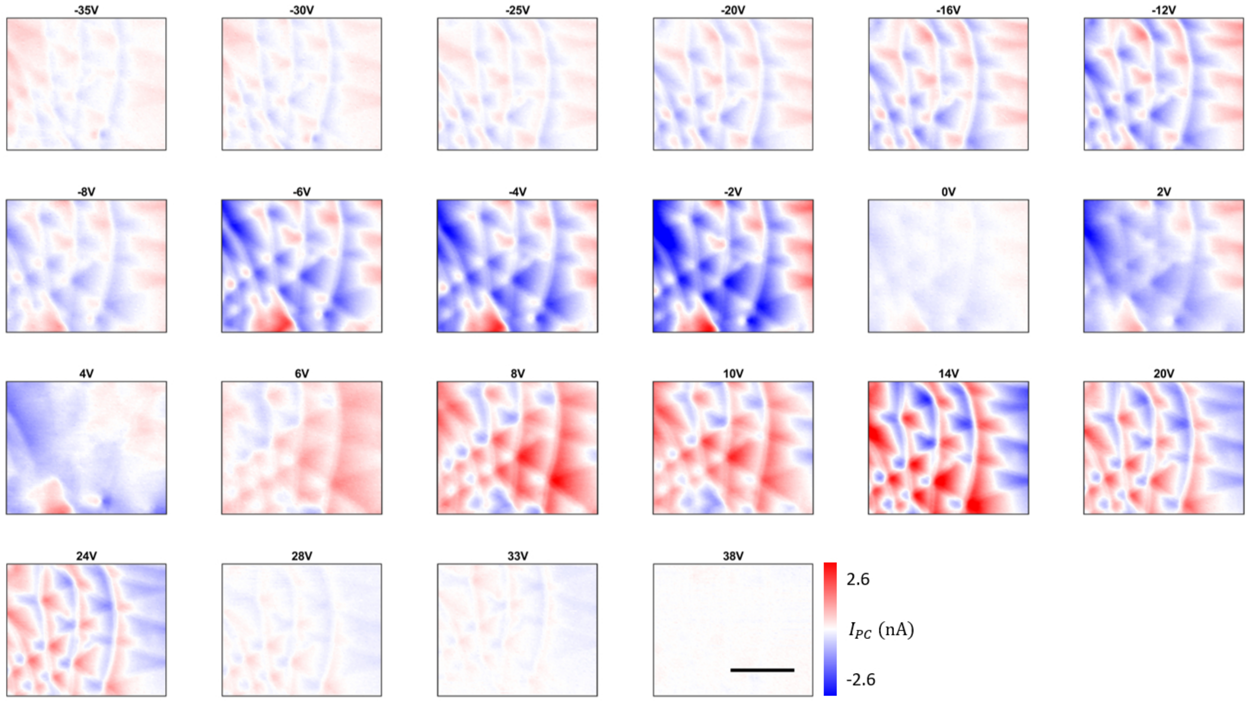

Supplementary Figure 3 | Photocurrent data for several gate voltages at $\omega = 900\text{cm}^{-1}$. Scale bar 1µm.

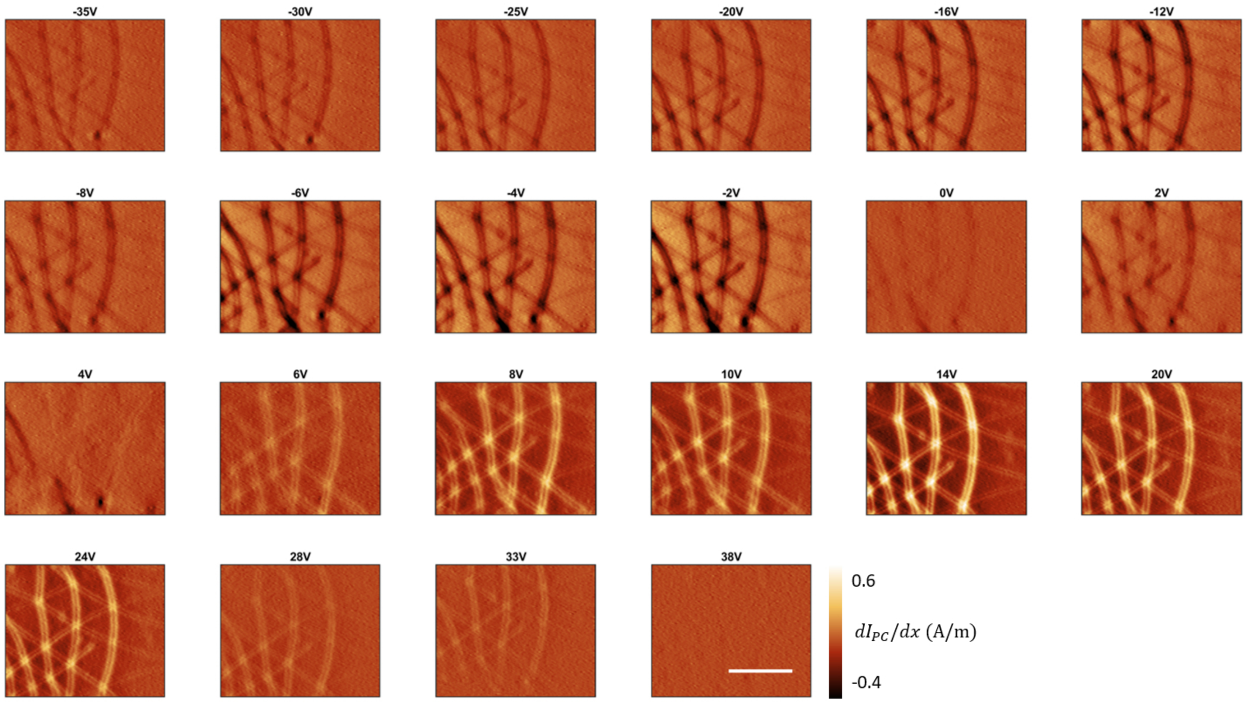

Supplementary Figure 4 | Photocurrent gradient for several gate voltages at $\omega = 900\text{cm}^{-1}$. Scale bar 1µm.

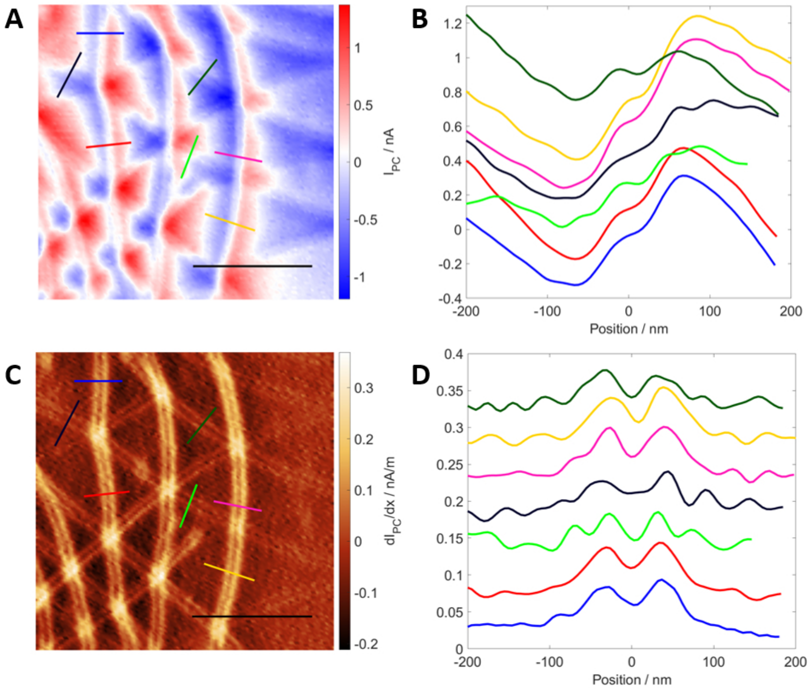

Supplementary Figure 5 | Photocurrent line profiles at $V_G = +24\text{V}$. (A) Nano-photocurrent image at $V_G = +24\text{V}$ (same as Figure 1(B) of main text). (B) Multiple line profiles across the domain walls. Each profile is offset by an arbitrary number for clarity. (C) and (D) same as (A) and (B) but for dI_{PC}/dx.

Supplementary Note 2.2: hBN reststrahlen band

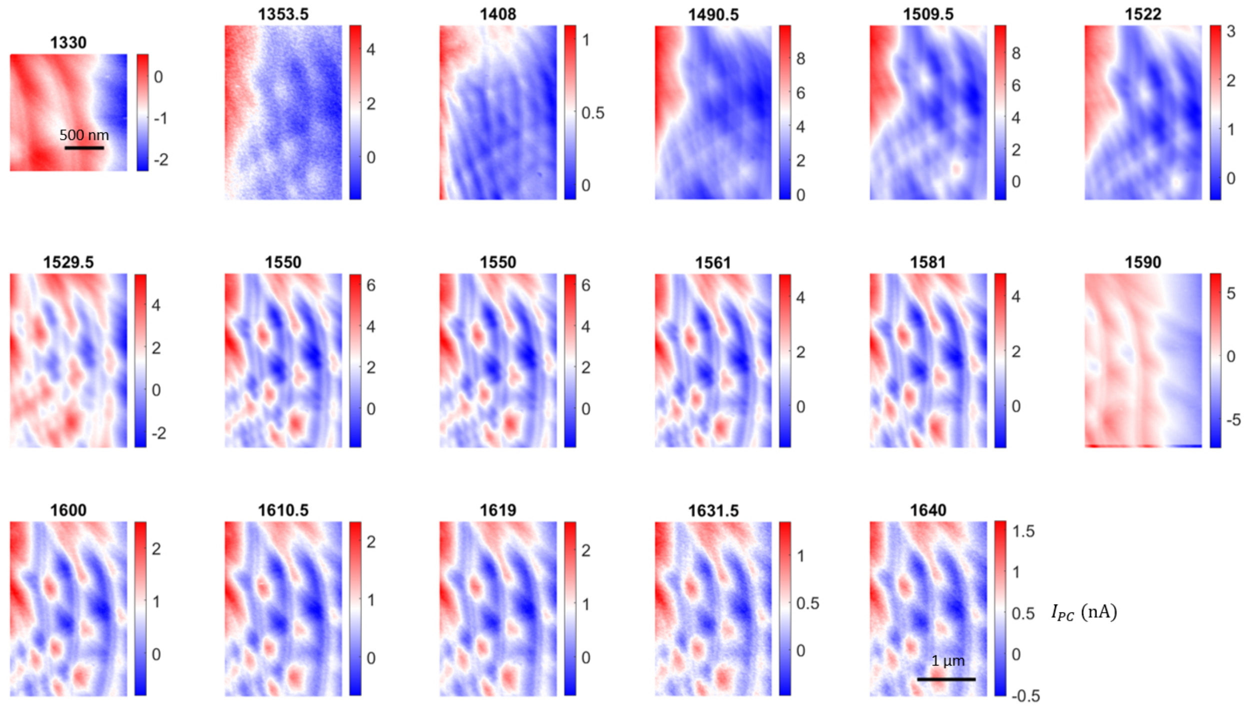

Supplementary Figure 6 | Full frequency dependent plots of the photocurrent in the hBN reststrahlen band at $V_G = +10\text{V}$.

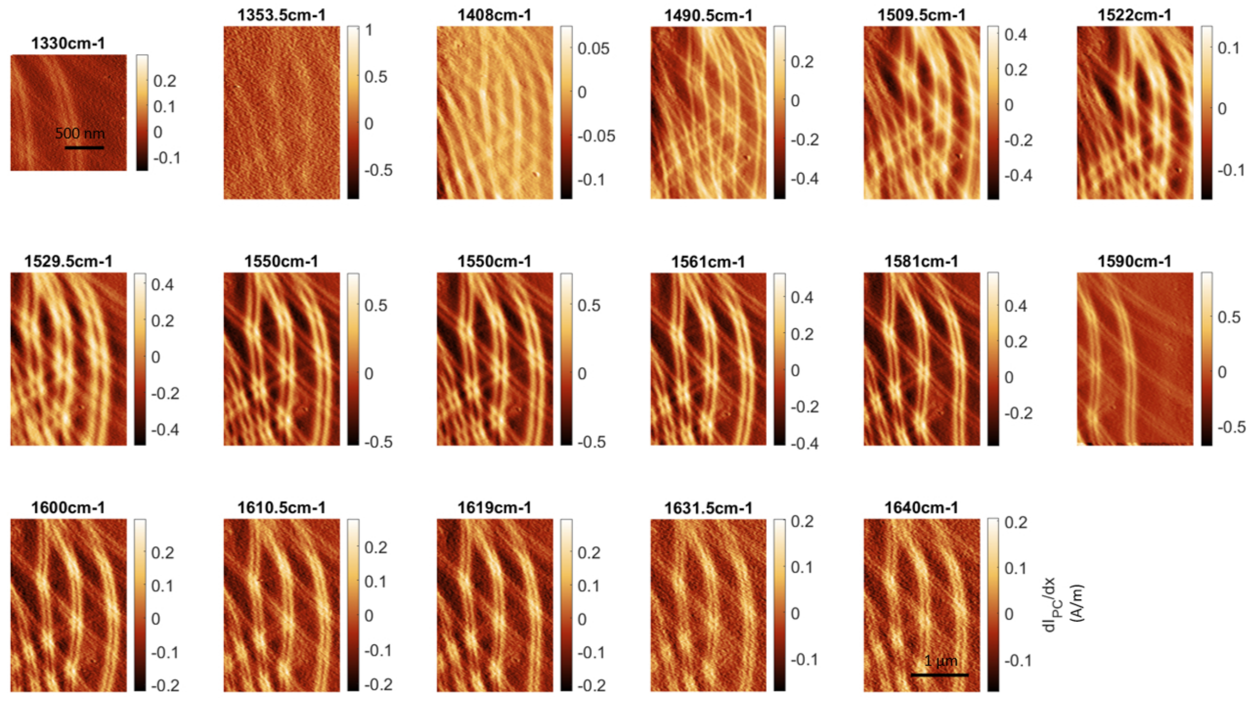

Supplementary Figure 7 | Frequency dependent plots of the gradient of the photocurrent in the hBN reststrahlen band at $V_G = +10\text{V}$.

Supplementary Note 3: Photocurrent model

Supplementary Note 3.1: Photocurrent calculation

In gapless materials such as graphene, the spatial photocurrent profiles are described by the Shockley-Ramo formalism (*2*). In this formalism, an auxiliary potential ϕ is defined as solution of Laplace's equation, $\nabla \cdot (\sigma^T \nabla \phi) = 0$ (σ is the dc conductivity tensor) with the contact configuration dependent boundary conditions: $\phi = 1$ at current collecting contacts (where the current is being measured) and $\phi = 0$ at the rest of the grounded contacts. According to the Shockley-Ramo formalism, one can show that the measured photocurrent would then be:

$$I_{PC} = \iint d^2\boldsymbol{r}' \boldsymbol{J}_{local}(\boldsymbol{r}') \cdot \nabla \phi(\boldsymbol{r}') \quad \text{(Eq 2)}$$

Where $\boldsymbol{J}_{local}$ is the locally generated photocurrent density. In our case the photocurrent is generated through the photothermoelectric effect, and for a tip positioned at a point $\boldsymbol{r}$ would therefore yield the following photocurrent reading:

$$I_{PC}(\boldsymbol{r}) = \iint d^2\boldsymbol{r}' \, \sigma(\boldsymbol{r}') \, S(\boldsymbol{r}') \, \nabla T(\boldsymbol{r}', \boldsymbol{r}) \cdot \nabla \phi(\boldsymbol{r}') \quad \text{(Eq 3)}$$

where $T(\boldsymbol{r}', \boldsymbol{r})$ is the temperature at $\boldsymbol{r}'$ as a result of a tip located at $\boldsymbol{r}$ and S is the Seebeck coefficient tensor.

Since we are interested in a 1D domain wall, we can simplify the problem with a quasi-1D geometry. We assume that the sample is infinite in the y direction, both σ and S are independent of y, and we have a grounded contact at $x = 0$ and a collecting contact at $x = L$. We further assume that S is diagonal and isotropic. These assumptions yield: $\phi(x) = \int_0^x dx' \frac{1}{\sigma(x')} / \int_0^L dx' \frac{1}{\sigma(x')}$. After substitution into the photocurrent expression we get:

$$I_{PC}(\boldsymbol{r}) = \frac{\Sigma}{L} \iint d^2\boldsymbol{r}' \, S(\boldsymbol{r}') \frac{\partial T(\boldsymbol{r}', \boldsymbol{r})}{\partial x} \quad \text{(Eq 4)}$$

Where $\Sigma \equiv L \left(\int_0^L dx' \frac{1}{\sigma_{xx}(x')} \right)^{-1}$. Finally, we assume the shape of the temperature profile to be independent of tip position, such that: $T(\boldsymbol{r}', \boldsymbol{r}) = T(\boldsymbol{r}' - \boldsymbol{r})$. This assumption is justified if the absorption and thermal properties are not strongly modulated as a function of position. The last assumption formulates the above expression for the measured photocurrent as a 2D convolution of two terms such that:

$$I_{PC}(\boldsymbol{r}) = \frac{\Sigma}{L} \left(S * \frac{\partial T}{\partial x} \right)(\boldsymbol{r}) \quad \text{(Eq 5)}$$

The remaining task in order to calculate the photocurrent is to calculate the temperature spatial profile, $T(\boldsymbol{r})$. We describe it by the diffusion equation:

$$-\kappa \nabla^2 \tau(\boldsymbol{r}) + g\tau(\boldsymbol{r}) = P(\boldsymbol{r}) \quad \text{(Eq 6)}$$

where $\tau = T - T_0$ is the electronic temperature change relative to a background thermal bath at T_0, κ is the in-plane thermal conductivity of graphene, g is the out-of-plane thermal coupling to the substrate (both assumed to be spatially uniform for simplicity) and P is the absorbed heat distribution (which is estimate in this study using the lightning rod model as described in Supplementary Note 3.3). Following (*3*), the general solution can be obtained by a Green's function approach, where we first solve for the Green's function G that satisfies the impulse response equation:

$$-\kappa \nabla^2 G(\boldsymbol{r}) + gG(\boldsymbol{r}) = \delta^{(2)}(\boldsymbol{r}) \quad \text{(Eq 7)}$$

where $\delta^{(2)}(\boldsymbol{r})$ is the 2D delta function. The general solution to Eq 6 for an arbitrary $P(\boldsymbol{r})$ is then given by the convolution $\tau = G * P$. We can solve for the Green's function through a Fourier analysis. We define $\tilde{G}(k_x, k_y) = \int_{-\infty}^{\infty} dx \int_{-\infty}^{\infty} dy\, G(x, y) e^{-i(k_x x + k_y y)}$ to be the Fourier transform of $G(x, y)$. One can then show that:

$$\tilde{G}(k_x, k_y) = \frac{1}{4\pi^2} \frac{1}{g + \kappa(k_x^2 + k_y^2)} \quad \text{(Eq 8)}$$

Taking the inverse Fourier transform gives us the Green's function

$$G(\boldsymbol{r}) = K_0\left(\frac{r}{\sqrt{\kappa/g}}\right) \quad \text{(Eq 9)}$$

where $K_0(x)$ is the 0th order modified Bessel function of the second kind and $l_{cool} = \sqrt{\kappa/g}$ is a thermal length-scale which is typically called the cooling length. In our simulations, we used $l_{cool} = 100\text{nm}$ for the room temperature $\omega = 900\text{cm}^{-1}$ data (Fig 2D) and $l_{cool} = 200\text{nm}$ for the $T = 200\text{K}$ data in the hBN Reststrahlen band (Figure 3C and 4B).

Assuming the graphene sheet thermal conductivity of $\kappa \sim 10^{-6}\ \text{W/K}$ (*4*) and a room temperature cooling length of $l_{cool} = 100\ \text{nm}$, the interfacial thermal resistance in our samples is about $R_T = 1/g = l_{cool}^2/\kappa \sim 10^{-8}\ \text{m}^2\text{K/W}$, comparable to theoretically predicted (*5*) and experimentally measured (*6*) values. Note that this parameter appears to strongly depend on the interface and sample quality. In a previous photocurrent experiment (*7*), this thermal resistance was estimated to be as high as $10^{-5}\ \text{m}^2\text{K/W}$.

The Shockley-Ramo formalism also provides an explanation for the asymmetry between the domain wall dI_{PC}/dx profiles along different directions in Figure 1. The profiles along the y-direction are significantly stronger because of the $\partial T/\partial x$ term in Eq 5. The domain walls along the other directions contribute less to the convolution in Eq 5 and therefore appear weaker in the experiment. This behavior is captured directly in Fig 2(D).

Supplementary Note 3.2: First principles calculations of Seebeck coefficient across the domain wall

We will analyze the static transport properties across a single AB/BA domain wall. The Hamiltonian is adopted from (*8*) where the optical properties across a single domain wall were discussed i.e., we consider the general Hamiltonian of bilayer graphene

$$H = \begin{pmatrix} H_0 & U^\dagger \\ U & H_0 \end{pmatrix}, U = \begin{pmatrix} U_{AA} & U_{AB} \\ U_{BA} & U_{BB} \end{pmatrix}, \quad \text{(Eq 10)}$$

where $H_0 = \hbar v_F \sigma \cdot \boldsymbol{k}$ denotes the Hamiltonian of a single layer graphene and U the interlayer coupling with $U_{AA} = U_{BB} = \frac{t_1}{3}\left[1 + 2\cos\left(\frac{2\pi}{3}\frac{\delta}{a_0}\right)\right], U_{AB} = \frac{t_1}{3}\left[1 + 2\cos\left(\frac{2\pi}{3}\left(\frac{\delta}{a_0} + 1\right)\right)\right], U_{BA} = \frac{t_1}{3}\left[1 + 2\cos\left(\frac{2\pi}{3}\left(\frac{\delta}{a_0} - 1\right)\right)\right]$ (*9*). A single AB-BA domain wall at $x = 0$ with width w is then modeled by the displacement field $\delta(x) = \frac{2}{\pi}\arctan\left[\exp\left(\frac{\pi x}{w}\right)\right] + 1$. For numerical convenience, we add another, independent, single BA/AB domain wall in order to implement periodic boundary conditions.

The particle current and heat-flow due to electrons is given by (*10*)

$$\begin{pmatrix} \vec{J} \\ \vec{U} \end{pmatrix} = \begin{pmatrix} \mathbf{K_0} & \mathbf{K_1} \\ \mathbf{K_1} & \mathbf{K_2} \end{pmatrix} \begin{pmatrix} e\vec{\nabla}\phi \\ T^{-1}\vec{\nabla}T \end{pmatrix} \quad \text{(Eq 11)}$$

where the tensors $\mathbf{K}_l$ with $l = 0, 1, 2$ read

$$\mathbf{K}_l = \frac{g_s g_v}{A} \sum_{\boldsymbol{k},n} \vec{v}_{\boldsymbol{k},n}\, \vec{v}_{\boldsymbol{k},n}^T \tau_{\boldsymbol{k},n} (\epsilon_{\boldsymbol{k},n} - \mu)^l \left(-\frac{\partial f_{\boldsymbol{k},n}^0}{\partial \epsilon_{\boldsymbol{k},n}}\right). \quad \text{(Eq 12)}$$

These quantities depend on the relaxation time $\tau_{\boldsymbol{k},n}$ and $\vec{v}_{\boldsymbol{k},n} = \langle \boldsymbol{k}, n | \hat{\vec{v}} | \boldsymbol{k}, n \rangle$ where $\epsilon_{\boldsymbol{k},n}$ and $|\boldsymbol{k}, n\rangle$ denote the eigenvalues and eigenvectors of the underlying Hamiltonian, respectively with $\boldsymbol{k}$ inside the first Brillouin zone. Furthermore, $f_{\boldsymbol{k},n}^0$ denotes the Fermi-Dirac distribution function at chemical potential μ, A denotes the area of the sample, $g_s = g_v = 2$ the spin and valley degeneracy, and $\hat{\vec{v}}$ is the velocity operator. Typical transport properties such as the dc conductivity, the Seebeck coefficient and the thermal conductivity are then defined by $\sigma_{dc} = e^2\mathbf{K}_0$, $S = -(eT)^{-1}\mathbf{K}_0^{-1}\mathbf{K}_1$, and $\kappa = T^{-1}(\mathbf{K}_2 - \mathbf{K}_1\mathbf{K}_0^{-1}\mathbf{K}_1)$.

Eq 11 can be generalized to define the local current response i.e., $\mathcal{J}(\vec{r}) = \int d\vec{r}'\mathcal{K}(\vec{r}, \vec{r}')\nabla\chi(\vec{r}')$ with $\mathcal{J}(\vec{r}) = \left(\vec{J}(\vec{r}), \vec{U}(\vec{r})\right)^T$ and the corresponding definitions for $\mathcal{K}(\vec{r}, \vec{r}')$ and $\chi(\vec{r})$. We then applied the local approximation (*11*) which amounts to $\mathcal{K}_{loc}(\vec{r}) = \int d\vec{r}'\mathcal{K}(\vec{r}, \vec{r}')$ and obtained the local transport quantities such as the Seebeck coefficient that were discussed in the main text.

Supplementary Note 3.3: Electric field profiles using the lightning rod model

The electric field relevant for calculating a temperature profile at the graphene layer is computed using the lightning rod of probe-sample near-field interaction (*12*). Here the near-field probe is considered as an ideally conducting metallic hyperboloid (roughly conical in shape) 19 microns in height with a taper angle of about 20 degrees to the probe axis, and a curvature radius of 75 nm at its apex. For a chosen sample configuration comprising a multi-layer stack (here a 7 nm top hBN layer, nearly charge-neutral graphene bilayer atop a 36 nm hBN slab over an SiO_2 substrate), a specified probe-sample distance *d,* and illumination energy, the model predicts the axisymmetric charge distribution $\lambda(z) \equiv dQ/dz$ along the probe. For the ideally conducting probe, this charge conforms to the external profile of the probe in a quasi-continuum of rings of radius $\mathcal{R}(z)$, where z denotes the probe's axial coordinate. From $\lambda(z)$, we evaluate the electric near-field from the probe in the graphene plane using the angular spectrum representation:

$$\boldsymbol{E_{probe}}(\rho, d) = \int_0^L dz\,\lambda(z) \int dq\, q\, [J_0(q\rho)\hat{z} + J_1(q\rho)\hat{\rho}]\, J_0(q\mathcal{R}(z)) e^{-q(d+z)} \qquad \text{(Eq 13)}$$

Here ρ denotes the in-plane radial coordinate from the probe axis, q is a Fourier momentum. As an integral sum of Bessel functions $J_1(q\rho)$, the radial field $E_{\rho,probe}$ presents a roughly "donut"-shaped in-plane distribution as shown in Fig. 4 of the main text. The total field inclusive of fields reflected from the sample is then given similarly by:

$$\boldsymbol{E_{total}}(\rho, d) = \int_0^L dz\,\lambda(z) \int dq\, q \begin{bmatrix} \left(1 + r_p(q)\right) J_0(q\rho)\hat{z} + \\ \left(1 - r_p(q)\right) J_1(q\rho)\hat{\rho} \end{bmatrix} J_0(q\mathcal{R}(z)) e^{-q(d+z)} \qquad \text{(Eq 14)}$$

Here $r_p(q)$ denotes the momentum-resolved Fresnel reflection coefficient for *p*-polarized fields computed for our heterostructure with a transfer matrix method.

We now turn our attention to the electric fields associated with generating the temperature profile relevant for the PTE underlying our photocurrent imaging. Since photocurrents were obtained at the $n = 2, 3$ harmonics of the probe tapping frequency Ω, the spatially-resolved distribution of thermal power deposited in the graphene at these harmonics is given by:

$$P_n(\rho) \approx \mathrm{Re}(\sigma)\, \left|E_{\rho,n}\right|^2 \qquad \text{(Eq 15)}$$

Here σ represents the optical conductivity of graphene and $E_{\rho,n}$ denotes the radially polarized total field demodulated at harmonic n:

$$E_{\rho,n}(\rho) \equiv \frac{\Omega}{\pi} \int_0^{2\pi/\Omega} dt\ \cos n\Omega t \cdot E_\rho(\rho, d = \cos \Omega t) \qquad \text{(Eq 16)}$$

Since the lightning rod model predicts a physically meaningful electric field profile for all probe-sample distances d, the power distribution $P_n(\rho)$ for $n = 2,3$ was straightforwardly calculated

with the relevant products of demodulated field distributions $E_{\rho,n}(\rho)$ inclusive of reflected fields from the sample.

Supplementary Figure 8 shows the field and temperature profiles for several frequencies. We note that the dT/dx profile is qualitatively similar to our observed photocurrent pattern. Let's say, the Seebeck profile is narrow compared to the dT/dx such that it can be approximated as a delta function. Then, from Eq 5, we see that the photocurrent profile will be identical to dT/dx. Therefore, we conclude that any Seebeck coefficient profile that is significantly narrower than the cooling length will produce a photocurrent pattern that is consistent with our experimental data.

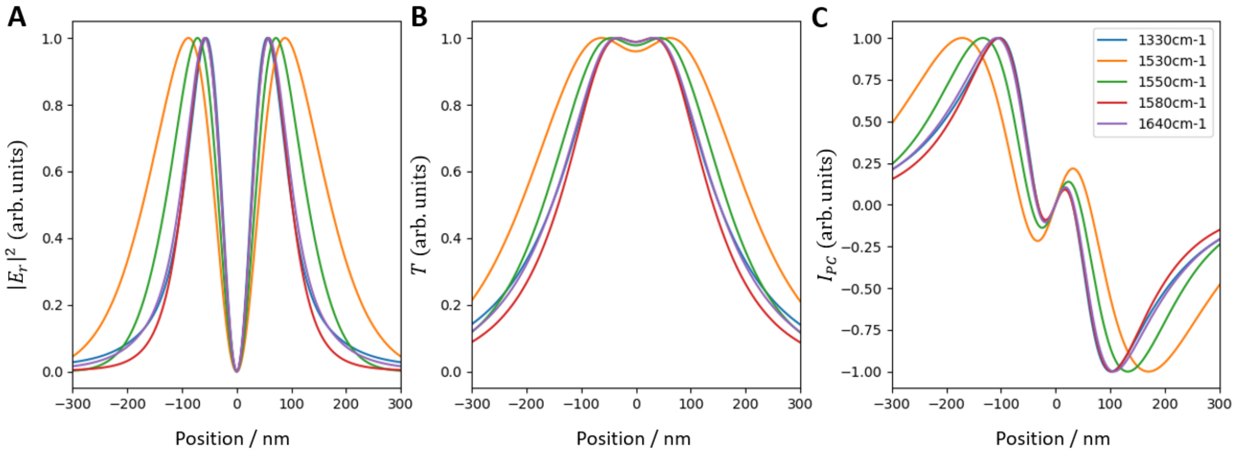

Supplementary Figure 8 | Electric field and temperature profiles. (A - C) Radial electric field E_r, hot carrier temperature T and $dT/dx = \hat{x} \cdot \nabla T$ profiles at various frequencies. The tip is located at the origin.

Supplementary Note 3.4: Electric field profiles using the point dipole model

In this section, we calculate the electric field and photocurrent profiles using a point dipole model.

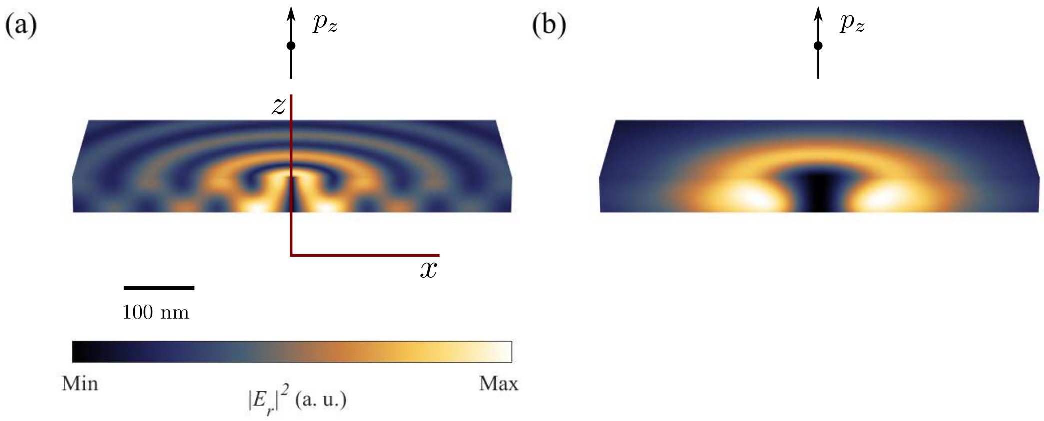

Supplementary Figure 9 | The absolute value of the in-plane electric field $E_r(x, y, z)$ created in the heterostructure by a vertically oriented dipole. Parameters: hBN thickness $d = 50\,\text{nm}$, frequency $\omega = 1530\text{cm}^{-1}$. (a) $z_{\text{tip}} = 40\text{nm}$, when multiple hot-rings are clearly visible. (b) $z_{\text{tip}} = 100\text{nm}$, when then $n = 1$ peak is dominant.

To obtain the photocurrent, we must first solve for the distribution of the electric field **E** in the system, which we model as a vacuum-hBN-SiO_2 multilayer. We ignore the weakly doped graphene layer (Fermi energy $E_F \sim 10\,\text{meV}$) encapsulated in hBN. Previous studies of similar nanostructures (S18) suggested that a small perturbation of the field distribution due to such a layer should not have a strong effect on the photocurrent. We choose the vacuum-hBN interface to be our x–y plane. We use ε_0 and ε_2 to denote the permittivities of the vacuum and the substrate, respectively. For hBN, whose in-plane and z-axis permittivities ε_1^t and ε_1^z are different, we define the effective permittivity $\varepsilon_1 = \sqrt{\varepsilon_1^t}\sqrt{\varepsilon_1^z}$. In the hyperbolic frequency region of hBN, ε_1 is almost purely imaginary, which plays the key role in the results, as we show below. Following the tradition in the literature, we simplify the problem by modeling the scanned probe (or "tip") as a dipole of magnitude $p^z\hat{\mathbf{z}}$, located at the point $(0, 0, z_{\text{tip}})$, see Supplementary Figure 9(a).

In the quasi-static approximation appropriate for near-field measurements, the electric field is given by $\mathbf{E} = -\nabla\Phi$ where $\Phi(\mathbf{r}, z)$ is the scalar potential and $\mathbf{r} = (x, y)$ is the in-plane position. The potential inside each medium can be deduced by considering transmission and reflection of the source dipole potential. For the upper half-space $z \geq 0$, we obtained the following expression:

$$\Phi(\mathbf{r}, z) = \varphi(r, z - z_{\text{tip}}) - \int \frac{d^2q}{(2\pi)^2} e^{i\mathbf{q}\cdot\mathbf{r}} r_P(q)\tilde{\varphi}(q, z + z_{\text{tip}}), \tag{17}$$

$$\tilde{\varphi}(q, z) = \frac{2\pi p^z}{\varepsilon_0} e^{-qz}, \quad \varphi(\mathbf{r}, z) = \int \frac{d^2q}{(2\pi)^2} e^{i\mathbf{q}\cdot\mathbf{r}} \tilde{\varphi}(q, z) = \frac{p^z}{\varepsilon_0} \frac{z}{\left(r^2 + z^2\right)^{3/2}}, \tag{18}$$

where $r_P(q)$ is the reflection coefficient (S19-S21)

$$r_P(q) = \frac{r_{01} - r_{21}e^{-iqz_0}}{1 - r_{01}r_{21}e^{-iqz_0}}, \qquad r_{ij} = \frac{\varepsilon_j - \varepsilon_i}{\varepsilon_j + \varepsilon_i}, \qquad z_0 = -2id\frac{\varepsilon_1}{\varepsilon_1^z}. \tag{19}$$

The integrand in Eq. (17) can be expanded in a power series of e^{-iqz_0}, i.e., as a sum of exponentials e^{-inqz_0} with $n = 0, 1, \ldots$, multiplied by constant coefficients. This means that it is possible to represent the potential as a sum over images of the source dipole. Such images can be thought to result from succesive reflections of the source potential from the two interfaces in the system. Integrating the series term by term, we obtain, for $z = 0$:

$$\Phi(r, 0) = (1 - r_{01})\varphi(r, z_{\text{tip}}) + (1 - r_{01}^2) r_{21} \sum_{n=1}^{\infty} (r_{01} r_{21})^{n-1} \varphi(r, z_{\text{tip}} + inz_0), \tag{20}$$

$$E_r(r, 0) = \frac{3p_z}{\varepsilon_0}(1 - r_{01}) \left[e_0(r) + (1 + r_{01}) r_{21} \sum_{n=1}^{\infty} (r_{01} r_{21})^{n-1} e_n(r) \right], \tag{21}$$

$$e_n(r) = \frac{(z_{\text{tip}} + inz_0) r}{\left[(z_{\text{tip}} + inz_0)^2 + r^2 \right]^{5/2}}. \tag{22}$$

Within the above multiple-reflection interpretaion, factor $r_{01} r_{21}$ in these series is the product of the reflection coefficients of the two interfaces.

Equation (21) is the desired expression for the total radial field E_r, which can be readily evaluated numerically. Representative examples are shown in Supplementary Figure 9 for $z_{\text{tip}} = 40\,\text{nm}$, $z_{\text{tip}} = 100\,\text{nm}$ and $d = 50\,\text{nm}$ for a frequency in the upper Reststrahlen band $\omega = 1530\,\text{cm}^{-1}$. In the first case, the field exhibits multiple maxima ("hot rings") along the radial direction, see Supplementary Figure 9(a). In the second case, there remains only one maximum. Supplementary Figure 9 also depicts the field distribution inside hBN. It reveals that the "hot rings" originate from directional rays bouncing between the two surfaces of the hBN layer, see Supplementary Figure 9(b). Such zigzag ray patterns in slabs of hyperbolic materials are well known from previous work (S18-S21). They are interpreted as trajectories of phonon-polariton collective modes undergoing a sequence of total internal reflections, as in the multiple-reflection picture of image formation introduced above.

In Supplementary Figure 10 we present $|E_r(r, 0)|^2$ calculated for a range of frequencies. The plot shows that as ω increases, the position of the field maximum stays nearly frequency-independent in the bottom part of the Reststrahlen band, then abruptly shifts to a larger r, then gradually moves back, returning to the original radial position once the upper edge of the Reststrahlen band is crossed. The frequency where the abrupt shift occurs gets larger when the dipole-sample separation z_{tip} gets smaller, cf. Supplementary Figure 10(a) and (b). Below we explain this behavior by analyzing relative importance of different terms in Eq. (21).

Consider a non-hyperbolic regime first. Here the images of order $n \geq 1$ are suppressed by powers of the factor $r_{01} r_{21}$ whose absolute value is less than unity. Additionally, the "vertical offsets" inz_0 in $e_n(r)$ [Eq. (22)] have the effect of broadening these functions in r by the amount $\text{Re}(inz_0) > 0$ and diminishing their maximum absolute values. As a result, the image series (21) converge quickly. For large probe-sample separations, $z_{\text{tip}} \gg iz_0$, we can approximate $e_n(r)$ by $e_0(r)$ and sum the resultant geometric series. In the opposite limit, $z_{\text{tip}} \ll iz_0$, we can simply drop the sum. In either case the total field has approximately the same profile $e_0(r)$ as the source dipole. This explains why in this regime only one maximum in $|E_r|^2$ occurs along the radial direction, at $r_0 \equiv z_{\text{tip}}/2$, Supplementary Figure 9(a).

For frequencies inside the Reststrahlen bands of hBN where it is hyperbolic, the field profiles are qualitatively different. In this case the factor $r_{01} r_{21}$ has the absolute value of unity if dissipation, i.e., the imaginary parts of the permittivities ε_1^t, ε_1^z, and ε_2 are neglected. Hence, powers of $r_{01} r_{21}$ do not suppress high-order terms in the series. They only add phase shifts in increments of $2\pi\alpha$ where α is defined by

$$\alpha = \frac{\ln r_{01} + \ln r_{21}}{2\pi i}. \tag{23}$$

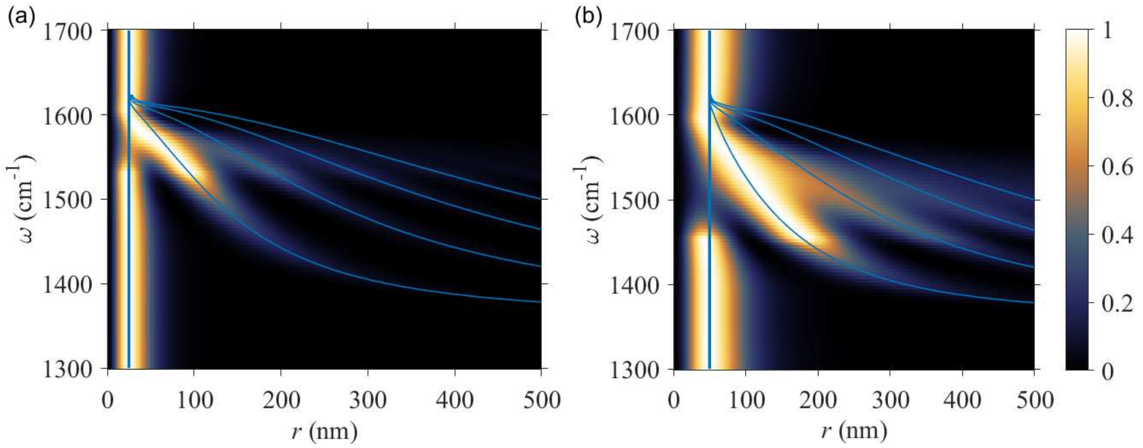

Supplementary Figure 10 | Pseudocolor plot of $|E_r|^2$ at the top hBN surface as a function of radial distance r and frequency ω. The vertical lines are at $r_0 = z_{\text{tip}}/2$. The curves indicate the peak positions $r_n = r_n(\omega)$ of image terms $1 \leq n \leq 4$ [Eq. (24)]. The electric field is normalized such that the maximum value of $|E_r|^2$ at each frequency is unity. Parameters: (a) $z_{\text{tip}} = 50\,\text{nm}$. (b) $z_{\text{tip}} = 100\,\text{nm}$. The hBN thickness is $d = 50\,\text{nm}$ in both panels.

Another important difference of the hyperbolic regime from the non-hyperbolic one is that z_0 becomes a real number (if the dissipation is again neglected). Therefore, instead of broadening, the vertical offsets inz_0 of the images in Eq. (22) mainly cause a radial shift in the position of the maxima of $|e_n|^2$. It is easy to see that these maxima are located at

$$r_n = \frac{1}{\sqrt{8}}\left[3(n^2 z_0^2 - z_{\text{tip}}^2) + \sqrt{25(n^4 z_0^4 + z_{\text{tip}}^4) + 14 n^2 z_{\text{tip}}^2 z_0^2}\,\right]^{1/2}. \tag{24}$$

In particular, $r_{n+1} - r_n \simeq z_0$ if $z_0 \gg z_{\text{tip}}$, see Supplementary Figure 11. It can be shown that the characteristic width of all $|e_n(r)|^2$ maxima is z_{tip}, and so these maxima are well separated in this small-z_0 regime. It is also easy to check that $|e_n(r_n)|^2$ monotonically decrease with n. Accordingly, the profile of $|E_r|^2$ contains a main peak near r_0 followed by a number of equidistant smaller peaks at $r \simeq r_n$. These are the aforementioned "hot rings." As z_0 drops with increasing frequency, the maxima of $|e_n(r)|^2$ move closer. They start to overlap more and more and eventually merge into a single peak.

Based on the above description, one may think that the global maximum of $|E_r|^2$ should be dictated by the $n = 0$ term, and so its position should remain close to r_0 at all frequencies. However, this is incorrect as evidenced by Supplementary Figure 9(b) and Supplementary Figure 10 where the $n = 1$ term is seen to dominate at some ω. To explain this phenomenon we can again invoke the multiple-reflection picture. When the polaritons bounce up and down inside the hBN layer, they experience total internal reflections characterized by the reflection coefficients r_{12} and $r_{10} = -r_{01}$ that are equal to unity by absolute value. However, the corresponding transmission coefficients t_{ij} are not zero. In fact, the absolute value of $t_{10} = r_{10} - 1 = -(1 + r_{01})$ is larger than unity unless the frequency is very near the upper edge of the hBN Reststrahlen band, $1604 < \omega(\text{cm}^{-1}) < 1625$. In other words, the field created by a polariton just outside hBN is typically enhanced compared to the field of a polariton incident on the interface from the inside. There is no problem with the energy conservation because this field becomes evanescent outside the hyperbolic medium. The factor $(1 + r_{01})r_{21}$ in Eq. (21) is precisely this enhancement factor. Boosted by it, the $n = 1$ term can dominate over the $n = 0$ one in an interval of ω where z_0 is of the order of z_{tip}.

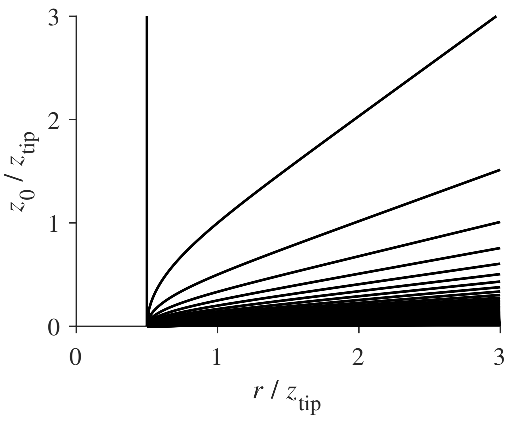

Supplementary Figure 11 | Positions r_n of the maxima in the partial fields $|e_n(r)|^2$ as functions of z_0 and $n = 0, 1, \ldots$ in the hyperbolic regime.

Indeed, Supplementary Figure 10 illustrates that the global maximum of the field stays close to r_0 (the vertical lines) at most frequencies yet there is a range of ω where the first-order image is more prominent and the global maximum shifts to r_1 (the first curve from the left). The ratio z_0/z_{tip} is between 1 and 2 in this range, so that $n = 0$ and $n = 1$ peaks are partially overlapping but distinct.

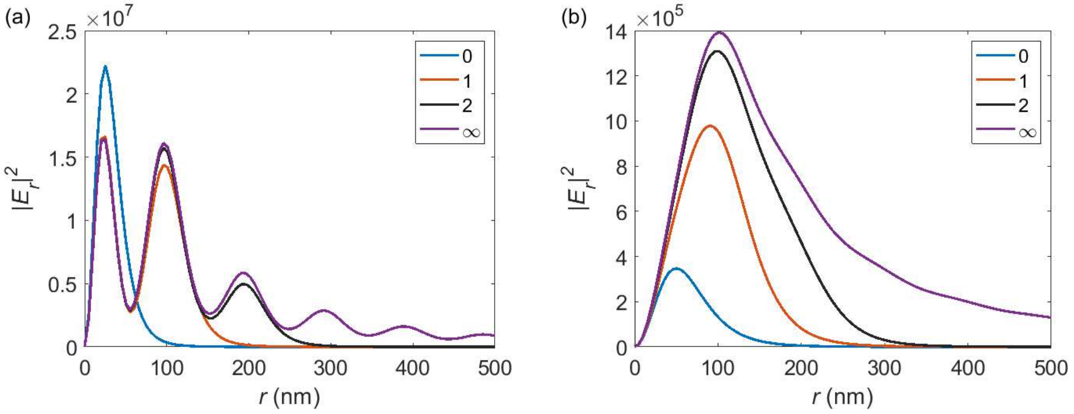

Supplementary Figure 12 | Profiles of $|E_r|^2$ (in arbitrary units) in the hyperbolic regime as a function of the number of images (indicated in the legend) retained. Parameters: $\omega = 1530\,\text{cm}^{-1}$, corresponding to $z_0 = (97 + 0.3i)\,\text{nm}$. (a) $z_{\text{tip}} = 50\,\text{nm}$. (b) $z_{\text{tip}} = 100\,\text{nm}$.

To further illustrate the competition between different n, we plot in Supplementary Figure 12 the results of partial summation of the image series. In particular, Supplementary Figure 12(a) depicts the case $z_0 \approx 2.5 z_{\text{tip}}$ where $n = 0$ and $n = 1$ peaks are still separate and approximately equal in strength. In Supplementary Figure 12(b) the nominal peak separation z_0 is approximately equal to z_{tip}. Here all the peaks have merged into one and the field profile appears to be smooth. The long-distance behavior of the total field in the latter regime is best understood by analyzing the Fourier integral in Eq. (17). For large r, it is governed by the poles of the reflection coefficient $r_P(q)$ at momenta

$$q_l = \frac{2\pi}{z_0}\,(l + \alpha)\,. \tag{25}$$

Here l is an integer and α is given by Eq. (23). These are the momenta of phonon-polariton eigenmodes of our hyperbolic slab (S18-S21). A short derivation shows that the contribution of lth eigenmode to the total field is given by

$$E_r^{(l)}(r) \simeq \pi \frac{p^z}{\varepsilon_0} \frac{q_l^2}{z_0} \frac{1 - r_{01}^2}{r_{01}} H_1^{(1)}(q_l r) e^{-q_l z_{\rm tip}}, \tag{26}$$

where $H_1^{(1)}(x)$ is the Hankel function. Under the condition $z_0 \ll z_{\rm tip}$ the principal mode $l = 0$, which has the smallest momentum q_0 is dominant; therefore,

$$|E_r|^2 \simeq |E_r^{(0)}(r)|^2 \propto \frac{e^{-2\,{\rm Im}\, q_0 r}}{r} e^{-2q_0 z_{\rm tip}}. \tag{27}$$

This behavior of $|E_r|^2$ is typical for weakly-damped waves induced by a local source in two dimensions. In the complete absence of dissipation, ${\rm Im}\, q_0 = 0$, our formula predicts the slow $1/r$ decay of $|E_r|^2$ at large distances from the origin, in agreement with Supplementary Figure 12. Strictly speaking, the field profile also contains small-amplitude oscillations superimposed on this $1/r$ tail due to the interference between the principal term $E_r^{(0)}(r)$ and a subleading $e_0(r) \propto 1/r^4$ term in $E_r(r)$ we did not include in Eq. (27).

In the next section we use the electric field profile as an input for computing photocurrent response. We show that for this purpose we need the line-integrated square of the field:

$$P_1(x) = \int dy |E_r(x,y)|^2. \tag{28}$$

According to Eq. (27), $P(r)$ decreases exponentially at large r, so that $P_1(x)$ is finite. In Supplementary Figure 13 we show $P_1(x)$ calculated numerically for the same parameters as in Supplementary Figure 12(b), e.g., $z_{\rm tip} = 100\,{\rm nm}$. The bottom curve in Supplementary Figure 13 includes only the $n = 0$ term in the images series, the next one $n = 0$ and 1, and so on. The top curve includes 10 terms, $0 \le n \le N = 9$. In all these cases, $P_1(x)$ has a minimum at the origin and a maximum at some positive x. The position of the maximum quickly approaches a limiting value as more terms in the series are retained. Increasing N also has the effect of making the decrease of $P_1(x)$ less steep at large x. From Eq. (26) we can conclude that this decay should be logarithmic, $P_1(x) \sim \ln|L_0/x|$ at $|x| < L_0 \equiv 1/({\rm Im}\, q_0)$ and more rapid at $|x| > L_0$. Having a finite N effectively places an upper cutoff $y \sim r_N \sim N z_0$ on the integral in Eq. (28). Therefore, while reproducing the position and shape of the maximum in $P_1(x)$ at small x requires only a handful of images, numerically exact result for the tail of $P_1(x)$ may necessitate up to L_0/z_0 images.

The value $z_{\rm tip} = 100\,{\rm nm}$ used in most of our numerical examples may seem somewhat large since the physical probe-sample distance in the experiment is typically less than 100 nm. This value of $z_{\rm tip}$ was chosen by treating it as a fitting parameter in the photocurrent simulations described in the next section. One should remember that the scanned probe is not a point dipole. Hence, $z_{\rm tip}$ is an effective probe-sample separation, which may indeed be relatively large if the tip was blunted or if sample inhomogeneity effectively broadened the statistically averaged field profiles. Note that for such $z_{\rm tip}$ the higher-order "hot rings" in the field distribution are either weak or non-existent. However, these additional maxima may potentially produce some effects when working with sharp tips or when the hot-ring separation matches the distance between adjacent domain walls. This may be an interesting subject for future study.

We now calculate the photocurrent profiles arising from the electric field profiles calculated above. Following Section S3.1, the photocurrent I_{PC} generated in the x-direction perpendicular to a single domain wall of infinite length centered at the y-axis is

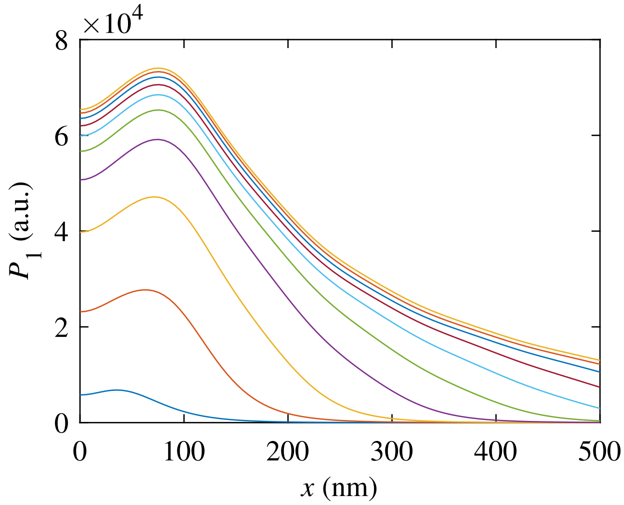

Supplementary Figure 13 | Function $P_1(x)$ [Eq. (28)], in arbitrary units. Different curves correspond to different number of terms $0 \leq n \leq N$ retained in the image series, from one ($N = 0$, bottom curve) to ten ($N = 9$, top curve). Parameters: same as in Supplementary Figure 12(b).

$$I_{PC}(x_{\rm tip}) = \frac{-1}{\sigma(0)} \int d^2 r_0 S(\mathbf{r}_0) \partial_x T(\mathbf{r}_0 - \mathbf{r}_{\rm tip}) \simeq A_1 T_1'(-x_{\rm tip}), \tag{29}$$

$$T_1(x) = \int dy\, [T(x,y) - T_0], \quad A_1 = \frac{-1}{\sigma(0)} \int dx_0 [S(x_0) - S(\infty)]. \tag{30}$$

To obtain the second equation in Eq. (29) we assumed that the local perturbation of the Seebeck coefficient with respect to the asymptotic value $S(\infty)$ occurs on length scales much shorter than the two characteristic scales of the temperature profile. Importantly, the line-integrated excess temperature $T_1(x)$ in Eq. (30) obeys the one-dimensional diffusion equation

$$-\kappa \partial_x^2 T_1(x) + g T_1(x) = P_1(x), \tag{31}$$

where $P_1(x)$ is the line-integrated power dissipation [Eq. (28)]. Solving Eq. (31) and substituting the solution into Eq. (29), we get, after some algebra:

$$I'_{PC}(x_{\rm tip}) = P_1(x_{\rm tip}) - \frac{1}{2l_c} \int dx e^{-\frac{|x - x_{\rm tip}|}{l_c}} P_1(x) \tag{32}$$

$$\simeq P_1(x_{\rm tip}) - \Delta P_1, \tag{33}$$

$$\Delta P_1 = \frac{1}{2l_c} \int dx e^{-\frac{|x|}{l_c}} P_1(x), \tag{34}$$

where we dropped the constant prefactor A_1/κ for simplicity. We also used the symmetry $P_1(-x) = P_1(x)$, which implies that the photocurrent gradient $I'_{PC}(x_{\rm tip})$ is an even function of $x_{\rm tip}$. Equations (32) and (33) are the key results of this section. The latter equation is valid for $|x_{\rm tip}| \ll l_c$.

If we neglect the second term in Eq. (33), we arrive at a simple approximate rule: the photocurrent gradient $I'_{PC}(x_{\rm tip})$ is proportional to $P_1(x_{\rm tip})$, the power dissipation in graphene integrated over the line $x_0 = x_{\rm tip}$ passing through the tip in the direction parallel to the domain wall. Actually, ΔP_1 may be a negligible correction because $P_1(x)$ has a long-range tail $P_1(x) \sim \ln|L_0/x|$ extending up to the phonon-

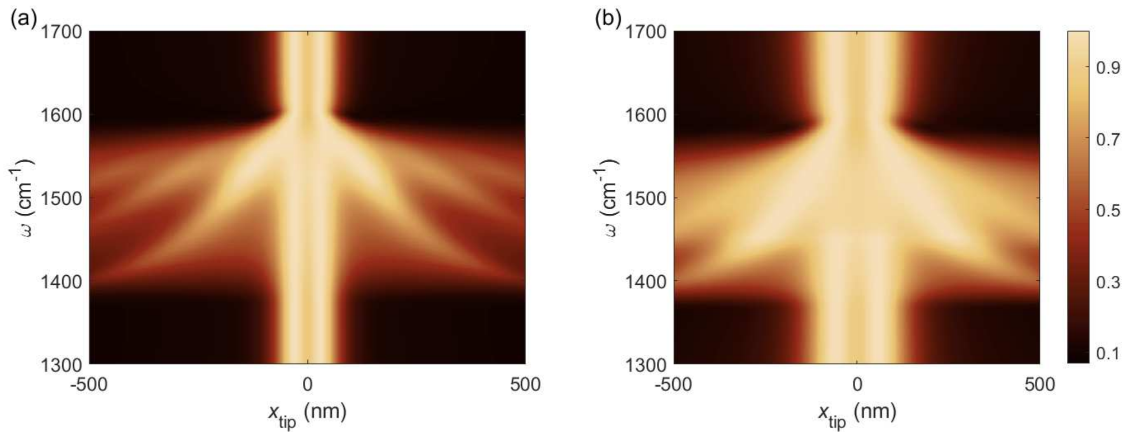

Supplementary Figure 14 | Positions of the extrema of the photocurrent gradient as a function of frequency for a cooling length $l_c = 1\mu$m for several values of z_{tip}. The photocurrent gradient is normalized by the maximum at each frequency, as in Supplementary Figure 10. (a) $z_{\text{tip}} = 50$ nm. (b) $z_{\text{tip}} = 100$ nm.

polariton decay length L_0, which may exceed l_c. However, even in that case ΔP_1 simply shifts I'_{PC} by a constant offset, similar to how different curves in Supplementary Figure 13 are shifted with respect to one another. This does not change the positions of interesting features of the photocurrent gradient such as its minima and maxima.

Numerical evaluation of Eq. (32) produces plots presented in Supplementary Figure 14. They show that $I'_{PC}(x_{\text{tip}})$ typically exhibits a minimum at $x_{\text{tip}} = 0$ and a maximum at some point $x_{\max} > 0$ and then a gradual decay at large x_{tip}, as seen in 3C of the main text. Depending on the ratio of z_{tip} to z_0, additional maxima may be observed, as seen in Supplementary Figure 14. Near the bottom of the Reststrahlen band or for smaller z_{tip}, these additional maxima become distinct, whereas for larger z_{tip} or for higher frequencies, the multiple peaks merge into one maximum. Our choice of z_{tip} reflects the absence of these additional “hot-rings" in the field and, by extension, the lack of observed secondary maxima in the photocurrent gradient. The positions of the maxima closest to the domain wall, $\pm x_{\max}$, which are present for all frequencies and values of z_{tip}, are determined by the field distribution, in particular, by the competition between r_0 and r_1, as discussed in the previous section.

Supplementary Note 3.5: Converting 1D profiles to 2D profiles – superposition model

To convert the 1D profiles calculated in Supplementary Note 3.2 into 2D profiles, we used a simple superposition model. However, the superposition model may not accurately reproduce the Seebeck profile at the AA sites. Here, we compare the relative importance of the domain walls and the AA sites to the calculated photocurrent pattern by separating their relative contributions.

First, we define a mask which is a series of Gaussians centered on the AA sites. Let the n AA sites be located at $\{x_n, y_n\}$. Then, the mask is given by

$$M(x,y) = \sum_n \exp\left(-\frac{(x-x_n)^2 + (y-y_n)^2}{w_{AA}^2}\right) \quad \text{(Eq 35)}$$

where w_{AA} is the width of the Gaussians. Then we separate the Seebeck coefficient at the AA sites by multiplying the Seebeck coefficient from the superposition model by the mask:

$$S_{AA}(x,y) = S_{2D}(x,y)M(x,y) \quad \text{(Eq 36)}$$

The domain wall contribution is then

$$S_{DW}(x,y) = S_{2D}(x,y)(1 - M(x,y)) \quad \text{(Eq 37)}$$

such that

$$S_{AA}(x,y) + S_{DW}(x,y) = S_{2D}(x,y) \quad \text{(Eq 38)}$$

Furthermore, since convolution is linear, the following is also true:

$$I_{PC,AA} + I_{PC,DW} = I_{PC} \quad \text{(Eq 39)}$$

where $I_{PC,AA}$, $I_{PC,DW}$ and I_{PC} are the photocurrent patterns arising from S_{AA}, S_{DW} and S_{2D} respectively.

Supplementary Figure 16 shows the Seebeck coefficient and photocurrent patterns arising from the profiles calculated above. We see that $I_{PC,AA}$ is simply a series of dipoles centered at the AA sites and does not resemble the pattern observed in the experiment. At the same time, $I_{PC,DW}$ reproduces both the meandering pattern as well as the fine features at the domain walls. The spatial patterns in the sum I_{PC} are only slight modifications to $I_{PC,DW}$. Therefore, we conclude that the 1D Seebeck coefficient variation across the domain wall is dominant in explaining the observed experimental pattern, thus justifying the use of the superposition model.

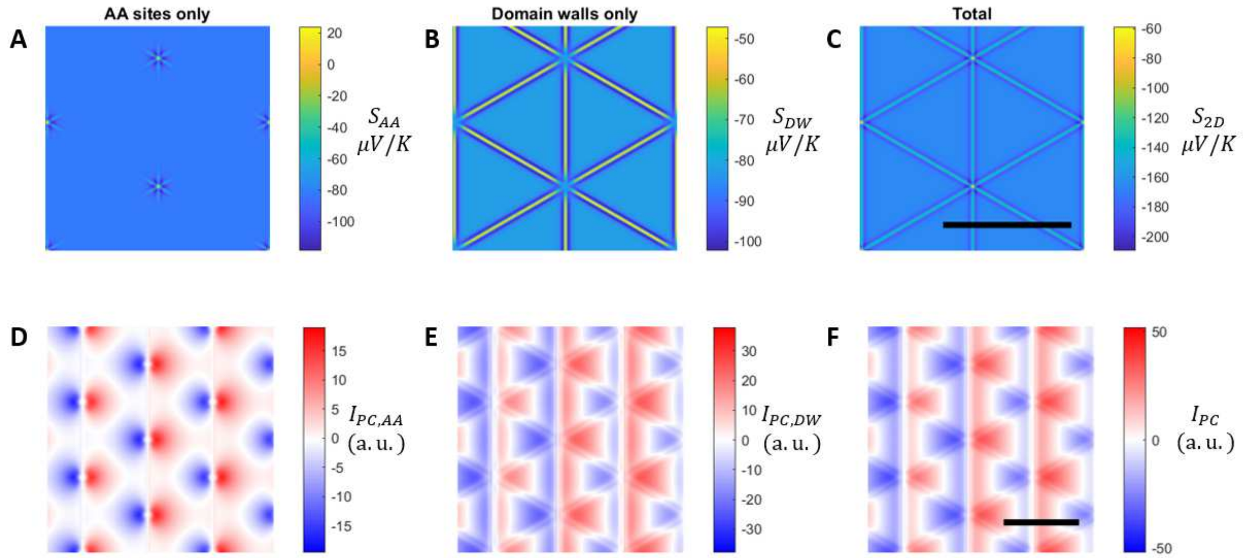

Supplementary Figure 16 | Relative importance of the AA sites and the domain walls to the calculated photocurrent pattern. (A) Seebeck coefficient of the AA sites only S_{AA} (B) Seebeck coefficient of the domain walls only S_{DW} (C) Total Seebeck coefficient calculated with the superposition model S_{2D}. (D – F) Calculated photocurrent patterns for the Seebeck coefficients in (A – C). Scale bars 500 nm.

Supplementary References

1. L. J. McGilly *et al.*, Visualization of moiré superlattices. *Nat. Nanotechnol.* **15**, 580–584 (2020).
2. J. C. W. Song, L. S. Levitov, Shockley-Ramo theorem and long-range photocurrent response in gapless materials. *Phys. Rev. B*. **90**, 075415 (2014).
3. A. Woessner *et al.*, Near-field photocurrent nanoscopy on bare and encapsulated graphene. *Nat. Commun.* **7**, 1–7 (2016).
4. A. A. Balandin *et al.*, Superior thermal conductivity of single-layer graphene. *Nano Lett.* **8**, 902–907 (2008).
5. R. Mao *et al.*, Phonon engineering in nanostructures: Controlling interfacial thermal resistance in multilayer-graphene/dielectric heterojunctions. *Appl. Phys. Lett.* **101** (2012), doi:10.1063/1.4752437.
6. Y. Liu *et al.*, Thermal Conductance of the 2D MoS2/h-BN and graphene/h-BN Interfaces. *Sci. Rep.* **7**, 1–8 (2017).
7. K. J. Tielrooij *et al.*, Out-of-plane heat transfer in van der Waals stacks through electron-hyperbolic phonon coupling. *Nat. Nanotechnol.* **13**, 41–46 (2018).
8. B. Y. Jiang *et al.*, Plasmon Reflections by Topological Electronic Boundaries in Bilayer Graphene. *Nano Lett.* **17**, 7080–7085 (2017).
9. M. Koshino, Electronic transmission through AB-BA domain boundary in bilayer graphene. *Phys. Rev. B*. **88** (2013), doi:10.1103/PhysRevB.88.115409.
10. J. M. Ziman, *Principles of the Theory of Solids* (Cambridge University Press, 1972; https://www.cambridge.org/core/product/identifier/9781139644075/type/book).
11. L. Brey, T. Stauber, L. Martín-Moreno, G. Gómez-Santos, Nonlocal Quantum Effects in Plasmons of Graphene Superlattices. *Phys. Rev. Lett.* **124**, 257401 (2020).
12. A. S. McLeod *et al.*, Model for quantitative tip-enhanced spectroscopy and the extraction of nanoscale-resolved optical constants. *Phys. Rev. B*. **90**, 085136 (2014).
13. A. Woessner *et al.*, Electrical detection of hyperbolic phonon-polaritons in heterostructures of graphene and boron nitride. *npj 2D Mater. Appl.*, 1–5 (2017).
14. S. Dai *et al.*, Subdiffractional focusing and guiding of polaritonic rays in a natural hyperbolic material. *Nat. Commun.* **6**, 1–7 (2015).
15. P. Li *et al.*, Hyperbolic phonon-polaritons in boron nitride for near-field optical imaging and focusing. *Nat. Commun.* **6**, 7507 (2015).
16. J.-S. Wu, D. N. Basov, M. M. Fogler, Topological insulators are tunable waveguides for hyperbolic polaritons. *Phys. Rev. B*. **92**, 205430 (2015).
